# SHANK2 mutations impair apoptosis, proliferation and neurite outgrowth during early neuronal differentiation in SH-SY5Y cells

**DOI:** 10.1038/s41598-021-81241-4

**Published:** 2021-01-22

**Authors:** Christine Unsicker, Flavia-Bianca Cristian, Manja von Hahn, Volker Eckstein, Gudrun A. Rappold, Simone Berkel

**Affiliations:** 1 Department of Human Molecular Genetics, Institute of Human Genetics, University Hospital Heidelberg, 69120 Heidelberg, Germany; 2 Department of Internal Medicine V, University Hospital Heidelberg, Heidelberg, Germany

**Keywords:** Neuroscience, Diseases of the nervous system, Genetics of the nervous system, Molecular neuroscience

## Abstract

*SHANK2* mutations have been identified in individuals with neurodevelopmental disorders, including intellectual disability and autism spectrum disorders (ASD). Using CRISPR/Cas9 genome editing, we obtained SH-SY5Y cell lines with frameshift mutations on one or both *SHANK2* alleles. We investigated the effects of the different *SHANK2* mutations on cell morphology, cell proliferation and differentiation potential during early neuronal differentiation. All mutant cell lines showed impaired neuronal differentiation marker expression. Cells with bi-allelic *SHANK2* mutations revealed diminished apoptosis and increased proliferation, as well as decreased neurite outgrowth during early neuronal differentiation. Bi-allelic *SHANK2* mutations resulted in an increase in p-AKT levels, suggesting that *SHANK2* mutations impair downstream signaling of tyrosine kinase receptors. Additionally, cells with bi-allelic *SHANK2* mutations had lower amyloid precursor protein (APP) expression compared to controls, suggesting a molecular link between *SHANK2* and APP. Together, we can show that frameshift mutations on one or both *SHANK2* alleles lead to an alteration of neuronal differentiation in SH-SY5Y cells, characterized by changes in cell growth and pre- and postsynaptic protein expression. We also provide first evidence that downstream signaling of tyrosine kinase receptors and amyloid precursor protein expression are affected.

## Introduction

Neurodevelopmental disorders have a high prevalence (1–3%) and are often caused by genetic factors. Genes of the *SHANK* (SH3 and multiple ankyrin repeat domains protein) family (comprising *SHANK1*, *SHANK2* and *SHANK3*) have been linked to a spectrum of neurodevelopmental disorders^1^. Deleterious de novo* SHANK2* mutations have been identified in individuals with intellectual disability (ID), autism spectrum disorders (ASD), developmental delay, and attention deficit and hyperactivity disorder^2–5^. In the SFARI gene database^6^ *SHANK2* is categorized as a high confidence autism risk gene. In addition, *SHANK2* has been linked to the pathology of neuropsychiatric (schizophrenia, bipolar disorder)^7–10^ and neurodegenerative disorders^11^.

*SHANK2* encodes for a postsynaptic scaffolding protein at glutamatergic synapses in the brain, essential for proper synapse formation, development and plasticity^12,13^. As organizer of a large protein complex, the so-called postsynaptic density, it connects the different types of glutamate receptors (α-amino-3-hydroxy-5-methyl-4-isoxazolepropionic acid receptor (AMPA), N-methyl-d-aspartat receptor (NMDA), metabotropic glutamate receptors) to molecules of many signaling pathways and to the actin cytoskeleton^14^. Impaired insulin signaling in the brain was postulated to contribute to the development of autism in genetically susceptible individuals^15^. As SHANK2 directly interacts with IRSp53 (insulin receptor substrate p53)^16,17^, it may be involved in insulin signaling in the brain, making this pathway susceptible to *SHANK2* mutations. In addition, studies in mice and human iPSC-derived neurons provided first evidence that a loss of SHANK could be partially rescued by administration of insulin like growth factor 1^18–20^. Moreover, SHANK dysregulation may also contribute to the molecular pathology of Alzheimer´s disease (AD)^21^. In synaptosomes isolated from the middle frontal gyrus from patients with AD increased SHANK2 levels were reported^11^. Mutations in the gene coding for amyloid precursor protein (APP) have been identified in several individuals with AD^22,23^. Both SHANK2 and APP are expressed during neuronal differentiation and are involved in synapse formation and neural plasticity^23–25^, and thus could potentially interact in the course of ASD and neurodegenerative disorders.

*SHANK2* gene variants have first been investigated in primary hippocampal cultures of rodents by overexpressing the respective mutated protein^5,26^. These analyses point to an impaired signal transduction at glutamatergic synapses, with a reduced synaptic density at dendrites^5,7^ and reduced AMPA receptors at the cell surface^26^, which are impairments that potentially underlie different neurodevelopmental disorders. *Shank2* knockout mice and rats have been generated, which show hyperactivity, repetitive behavior as well as social and cognitive impairments together with impaired synaptic transmission^27–30^. Recently, ASD-related *SHANK2* mutations were investigated in induced pluripotent stem cell-derived cortical neurons, reporting increased dendritic length and complexity as well as a perturbation of the expression of neurodevelopmental genes^31^. Further information about the functional impact of *SHANK2* mutations in humans is needed for a better understanding of the molecular pathology of neurodevelopmental disorders.

In this study we employed the human neuroblastoma cell line SH-SY5Y to model *SHANK2* variants. This cell line is a frequently cited in vitro model in neuropsychiatric research. Despite its tumor origin, its neuro-ectodermal lineage allows the investigation of neuronal phenotypes in the context of neurodevelopmental and neurodegenerative diseases^32^. SH-SY5Y cells express all three members of the *SHANK* gene family (*SHANK1, SHANK2, SHANK3*) and have been used to investigate the influence of sex hormones on SHANK expression^33^. Furthermore, the cells can be differentiated into neurons to model early neurodevelopmental processes of cortical development^32^, which is relevant as cortical function is central in ASD pathology^34,35^. Findings in this cellular model nevertheless should also be reproduced in a more physiological model, in order to strengthen the respective findings.

The aim of this study was to elucidate the impact of mono- and bi-allelic *SHANK2* frameshift mutations on different cellular properties during early neuronal differentiation by analyzing cell proliferation, neurite morphology, as well as neuronal and glutamatergic marker gene/protein expression. In addition, we explored the effect on receptor tyrosine kinase downstream signaling and on amyloid precursor protein (APP) expression to further improve our understanding of SHANK2 function.

## Results

### Genome editing of SH-SY5Y cells to generate *SHANK2* mutant lines

To investigate the functional consequences of *SHANK2* mutations that have been identified in patients with ASD and intellectual disability, we introduced *SHANK2* frameshift mutations in SH-SY5Y cells using CRISPR/Cas9-based genome editing. A guide RNA was designed to target the genomic region adjacent to the position of the *SHANK2*-R841X variant, which had previously been identified in a patient with ASD^4^. The non-homologous end-joining repair mechanism of the cells led to the introduction of a heterozygous adenosine insertion, which caused a frameshift and premature stop codon (6H6, p.L837Sfs*49). Additionally, we generated a compound heterozygous mutation line (6A4) with an adenosine deletion on one allele and an adenosine insertion on the other, resulting in two different premature stop codons (p.L837Sfs*49, p.L837Wfs*14) (Table 1). As a control, we selected cells that underwent the complete process of genome-editing without being edited (1F11) as well as the original SH-SY5Y line (WT). Mutations were confirmed after several passages (Supplementary Fig. 1) and putative predicted off-target sites were ruled out by Sanger sequencing of the respective genomic regions (Supplementary Table 1).

**Table 1:** Overview of genome-edited lines and controls.

Cell line	Genome (ENST00000338508.4)	Protein Q9UPX8
1F11	Wildtype	Wildtype
6A4	Compound heterozygous Chr11:70336426_70336427InsA Chr11:70336426_70336426DelA	p.L837Sfs*49 p.L837Wfs*14
6H6	Heterozygous Chr11:70336426_70336427InsA	p.L837Sfs*49

**Figure 1:**
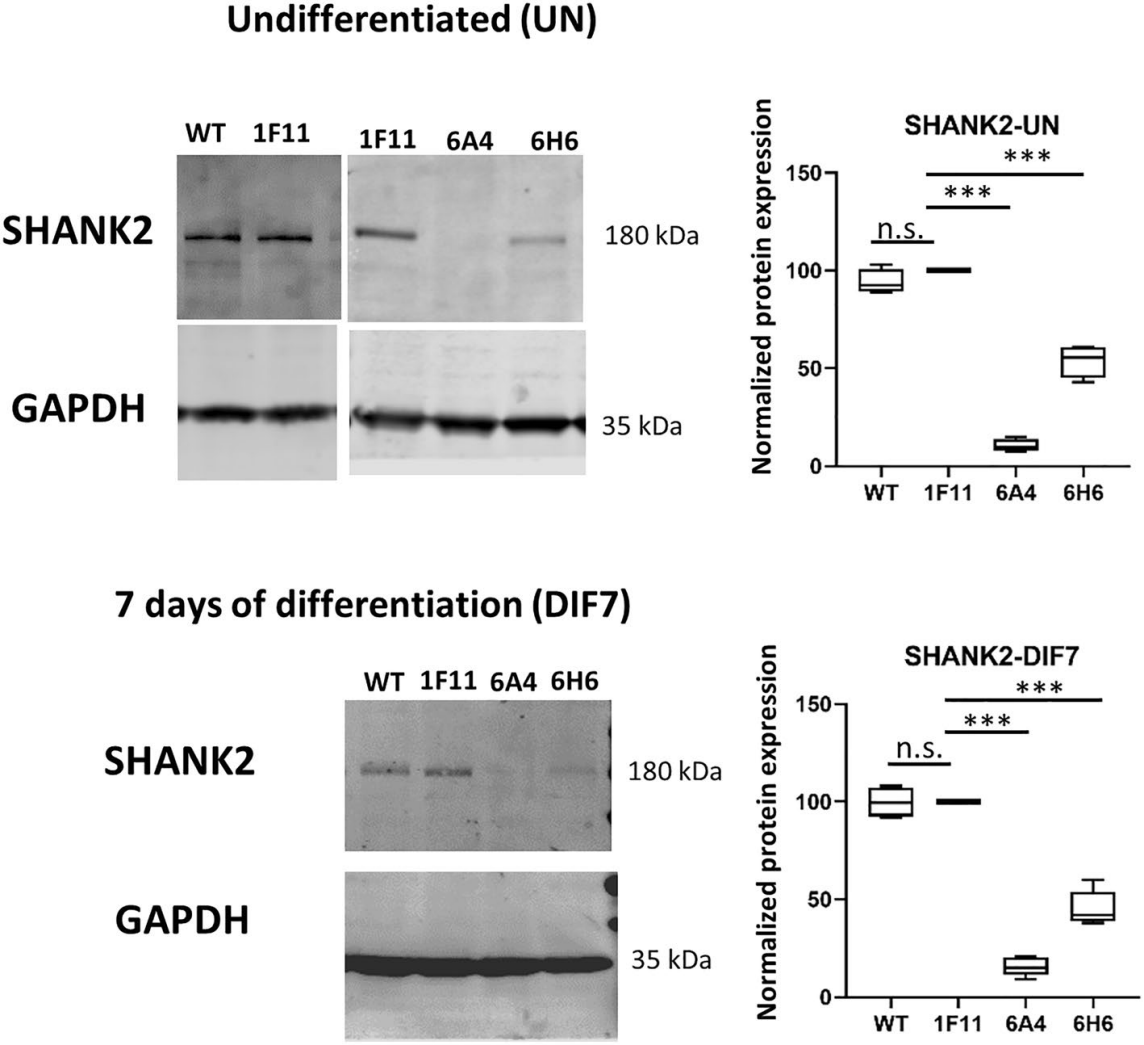
Western blot analysis of SHANK2 expression in genome-edited cell lines (6A4, 6H6) versus the non-edited controls (1F11, WT). SHANK2-expression was normalized against the expression of GAPDH (reference) in undifferentiated cells (UN) and in cells after 7 days of neuronal differentiation (DIF7). Cropped western blot membrane images are shown and full-length blots are presented in Supplementary Fig. 2. n = 4 experiments. One-way ANOVA was performed and the two different mutant cell lines as well as the WT control were compared to the control 1F11 (ANOVA results: SHANK2-UN F = 266.8, P < 0.0001; SHANK2-DIF7 F = 229.8, P < 0.0001). Correction for multiple testing with Dunnett’s test (n = 3 tests for each time point). Data were presented as box-plots and the corrected P-values are indicated. ***P ≤ 0.001.

Western blot experiments revealed a complete loss of SHANK2 wildtype expression for the compound heterozygous cell line and a reduction of SHANK2 wildtype protein by one half for the heterozygous *SHANK2* mutant lines (Fig. 1, Supplementary Fig. 2). The SHANK2 antibody targets amino acids 849–1029, a region downstream of the introduced frameshift mutation (6A4: p.L837Sfs*49, p.L837Wfs*14, 6H6:p.L837Sfs*49), therefore the reduction in protein levels reflects the diminished amount of wildtype SHANK2 protein, whereas the presence of a truncated version of the protein cannot be excluded. Treatment with cycloheximide, which blocks nonsense-mediated mRNA decay (NMD), did not reveal any evidence for NMD in both cell lines (Supplementary Fig. 3) even though quantitative real-time PCR (qPCR) analysis of *SHANK2* mRNA expression revealed significantly reduced levels in the cell line with compound heterozygous *SHANK2* mutations (Supplementary Fig. 4A).

### Cells with bi-allelic *SHANK2* mutations show increased proliferation and lower apoptosis rates compared to controls during neuronal differentiation

**Figure 2:**
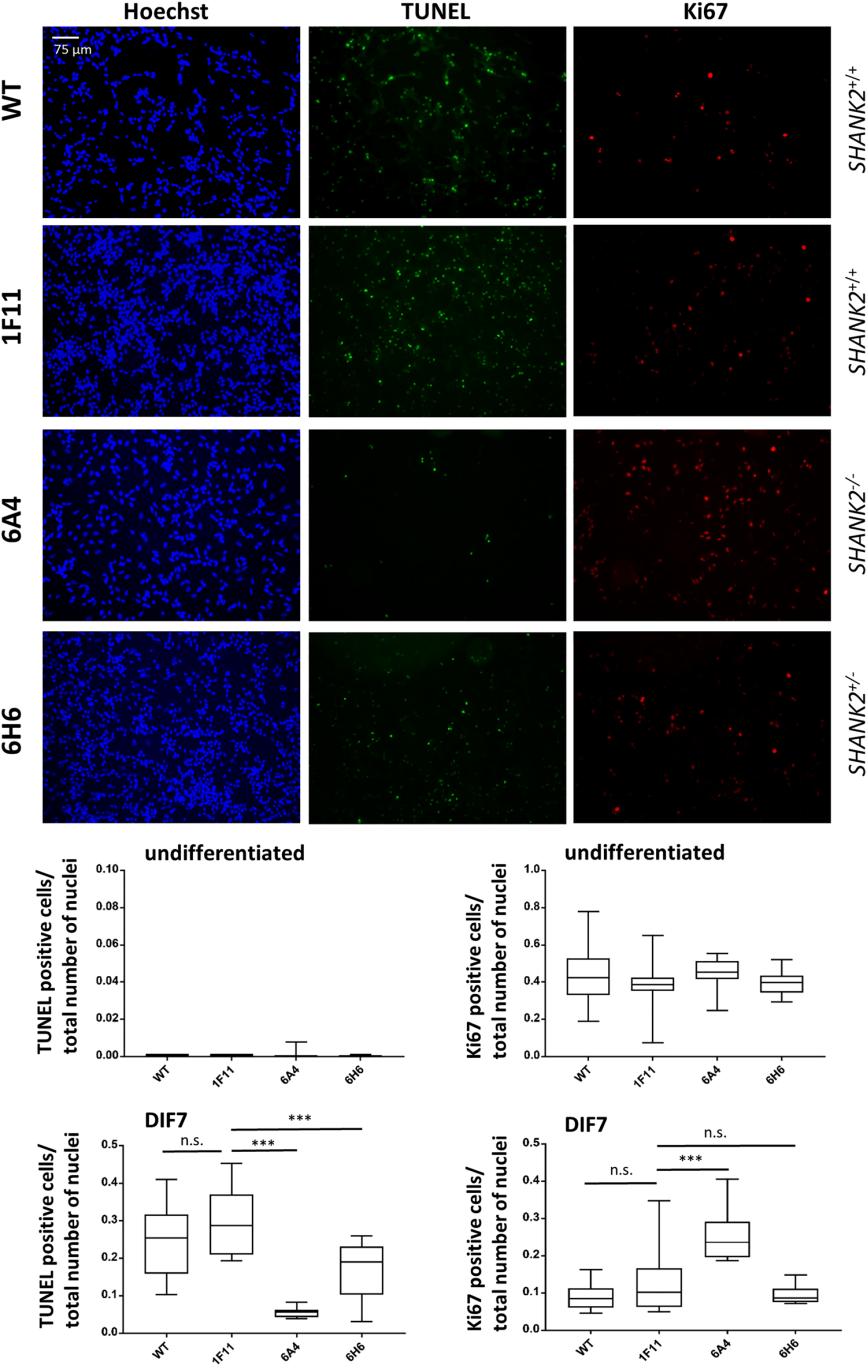
Analysis of apoptosis and cell proliferation in *SHANK2* mutant cell lines. A TUNEL assay was carried out to determine the rate of apoptotic cells and TUNEL-positive cells were quantified relative to the total number of nuclei (Hoechst), revealing significantly lower numbers of TUNEL-positive cells for both SHANK2 mutant lines compared to the control 1F11 (one-way ANOVA, F = 22.74, P < 0.0001, P-values after correction for multiple testing with Dunnett’s test 6A4 P < 0.0001, 6H6 P = 0.0004, WT P = 0.326) after 7 days of differentiation (DIF7). In addition, immunofluorescence microscopy was carried out with the proliferation marker Ki67 followed by quantification of the Ki67 positive cells versus the total number of nuclei. A significantly increased number of Ki67-positive cells was identified in cell line 6A4 with bi-allelic *SHANK2* mutation in comparison to the control 1F11 (one-way ANOVA, F = 20.97, P < 0.0001, P-values after correction for multiple testing with Dunnett´s test 6A4 P < 0.0001, 6H6 P = 0.49, WT P = 0.327) at DIF7. The pictures illustrate the expression after 7 days of differentiation. No difference was observed between the two control lines WT and 1F11. Undifferentiated cell lines showed equally low apoptosis levels (one-way ANOVA, F = 2.285, P = 0.09, corrected P-values Dunnett´s test 6A4 P = 0.078, 6H6 P = 0.98, WT P = 0.99) and equal levels of cell proliferation (one-way ANOVA, F = 1.12, P = 0.3478, corrected P-values Dunnett´s test 6A4 P = 0.30, 6H6 P = 0.99, WT P = 0.40). Data were presented in box plots. n = 3 experiments. ***P ≤ 0.001.

To assess basic properties of the *SHANK2*-mutant cell lines, we analyzed cell proliferation at the undifferentiated stage. Immunofluorescence microscopy with Ki67, a cell proliferation marker, showed similar proliferation rates for all lines. Apoptosis was analyzed with the TUNEL assay, which revealed low apoptosis rates under basic conditions without any differences between the cell lines (Fig. 2).

Next, we differentiated the SH-SY5Y lines according to an improved neuronal differentiation protocol published by Chiocchetti et al.^32^. With this protocol, SH-SY5Y cells can be differentiated into neurons that express genes relevant in early cortical development and associated to neurodevelopmental/-psychiatric disorders^32^. To validate the process of neuronal differentiation, we quantified the expression of selected marker proteins by Western blot analysis in the control cell line (WT) under basic culturing conditions and after 7 days of differentiation. A significant increase of the neuronal marker β3-Tubulin (TUBB3) together with decreased levels of the neuronal stem cell protein Nestin (NES) after 7 days of differentiation indicate that the SH-SY5Y cells are starting to differentiate into neurons (Supplementary Figs. 5 and 6). This finding is also supported by an increased expression of the presynaptic marker protein Synaptophysin (SYP) and the postsynaptic density protein 95 (PSD-95) (Supplementary Figs. 5 and 6). Expression analysis of AKT Serine/Threonine Kinase 1 (AKT) was also included, as it is an important mediator of growth factor-induced neuronal survival in the developing nervous system. No significant expression differences could be found for AKT, SHANK2 and the ionotropic glutamate receptor NMDA type subunit 2B (GRIN2B) (Supplementary Figs. 5 and 6).

As we could detect elevated protein levels of glutamatergic (SYP, PSD-95) and pan-neuronal (TUBB3) proteins in parallel to the reduction of the neuronal stem cell marker Nestin after 7 days of differentiation, we selected this stage for further analysis to investigate impairments in early neuronal differentiation. We analyzed cell proliferation after 7 days of differentiation when wildtype cells still proliferate with low apoptosis rates. The cell line 6A4 with bi-allelic *SHANK2* mutation showed significantly more Ki67-positive cells, indicating a higher rate of cell proliferation compared to controls, whereas the heterozygous line 6H6 behaved similarly to the controls (Fig. 2). However, reduced numbers of TUNEL-positive cells were identified for both cell lines with *SHANK2* mutations (6A4 and 6H6), indicating a lower apoptosis rate compared to controls (Fig. 2).

### Bi-allelic *SHANK2* mutation impairs neurite outgrowth

**Figure 3:**
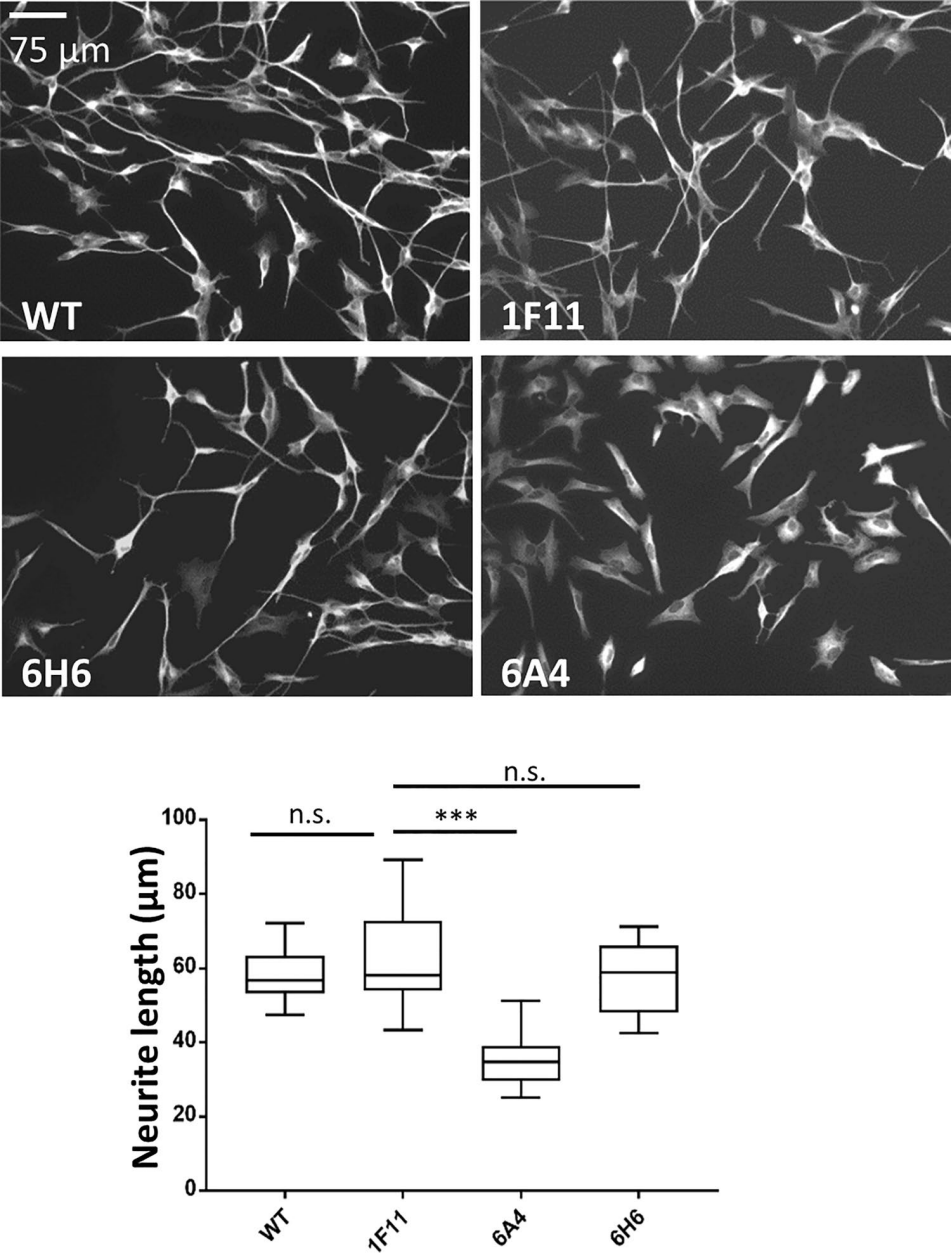
Analysis of cell morphology. Immunofluorescence microscopy was carried out with β3-Tubulin and the length of neurites after 4 days of differentiation was measured, revealing significantly shorter neurites for the cell line with compound heterozygous *SHANK2* mutation (6A4) in comparison to the control 1F11. n = 3 experiments, one-way ANOVA (F = 31.1, P < 0.0001), corrected P-values from Dunnett’s test (6A4 P < 0.0001, 6H6 P = 0.36, WT P = 0.48). No difference was observed between the two control lines WT and 1F11. Data were presented in box plots. ***P ≤ 0.001.

To identify morphological impairments in early neuronal differentiation processes, we analyzed neurite outgrowth after 4 days of differentiation in the different cell lines by measuring the length of β3-Tubulin-positive cellular extensions in FiJi^36^. The control cells (WT, 1F11) extended long neurites and started to form neuronal networks (Fig. 3). The heterozygous *SHANK2* mutant line 6H6 developed similarly to the controls, whereas the compound heterozygous *SHANK2* mutant line (6A4) showed an obvious impairment regarding network formation and neurite extension. The 6A4 cell line had significantly shorter neurites compared to controls and 6H6 (Fig. 3).

### Changes of gene expression in *SHANK2* mutant cells

To further elucidate the influence of *SHANK2* mutations on basic cellular and neuronal differentiation properties, we analyzed mRNA expression levels of several genes involved in these processes by qPCR (n = 3 experiments) (Supplementary Fig. 4). The bi-allelic mutation of *SHANK2* in SH-SY5Y cells initially led to strongly reduced SHANK2 mRNA levels compared to the control in the undifferentiated state. After 7 days of differentiation, however, *SHANK2* mRNA levels were equal between the cell lines and after 16 days of differentiation even an increase of *SHANK2* mRNA expression was found for the compound heterozygous cell line (Supplementary Fig. 4). We also analyzed the gene expression of *microtubule-associated protein 2* (*MAP2*), the presynaptic gene *synaptophysin* (*SYP*) and the postsynaptic gene *discs large MAGUK scaffold protein 4* (*DLG4*, encoding for PSD-95) to determine if neuronal, presynaptic and postsynaptic genes are differentially expressed. Furthermore, the expression of all *SHANK* family members (*SHANK1, SHANK2, SHANK3*) was analyzed as well as several glutamate receptor genes, as SHANKs are known to interact with glutamate receptors: *AMPA receptor subunit 2* (*GRIA2*), *metabotropic glutamate receptor 1* (*GRM1*) and *the ionotropic glutamate receptor kainate type 2* (*GRIK2*). To find out if GABAergic neurons or aminergic neurons are affected we included *GABA receptor beta 3* (*GABRB3*) and *thyroxin hydroxylase* (*TH*) expression analysis. The neuronal stem cell gene *Nestin* (NES) has been included to determine the presence of very immature neuronal cells.

In undifferentiated cells, a significantly increased expression of *NES* and a nominally significant reduction in the expression of both *SHANK1* and *SHANK2* was observed in the compound heterozygous line 6A4 (Supplementary Fig. 4A). Furthermore, expression of *GRIA2* and *GABRB3* were reduced. A gene predominantly expressed in aminergic neurons, *tyrosine hydroxylase*, was expressed at low levels with slightly elevated levels in the heterozygous cell line 6H6. *Nestin* levels remained robustly elevated in the cell line with a bi-allelic *SHANK2* mutation (6A4) from the undifferentiated state throughout neuronal differentiation after 7 and 16 days (Supplementary Fig. 4A–C). The results reached statistical significance after correction for multiple testing. Additionally, we found reduced levels of *tyrosine hydroxylase* in the compound heterozygous cell line (6A4) after 7 and 16 days of differentiation (Supplementary Fig. 4B,C).

After 16 days of differentiation, increased expression levels of the presynaptic gene *SYP*, all three members of the *SHANK* family as well as *GRIA2* and *GABRB3* were identified in the compound heterozygous *SHANK2* mutant line (Supplementary Fig. 4). No mRNA expression differences in *SHANK2* mutant lines were detected, neither for *DLG4*, *GRM1*, *GRIK2*, nor *MAP2* (Supplementary Fig. 4C).

### Changes of protein expression in *SHANK2* mutant cells

**Figure 4:**
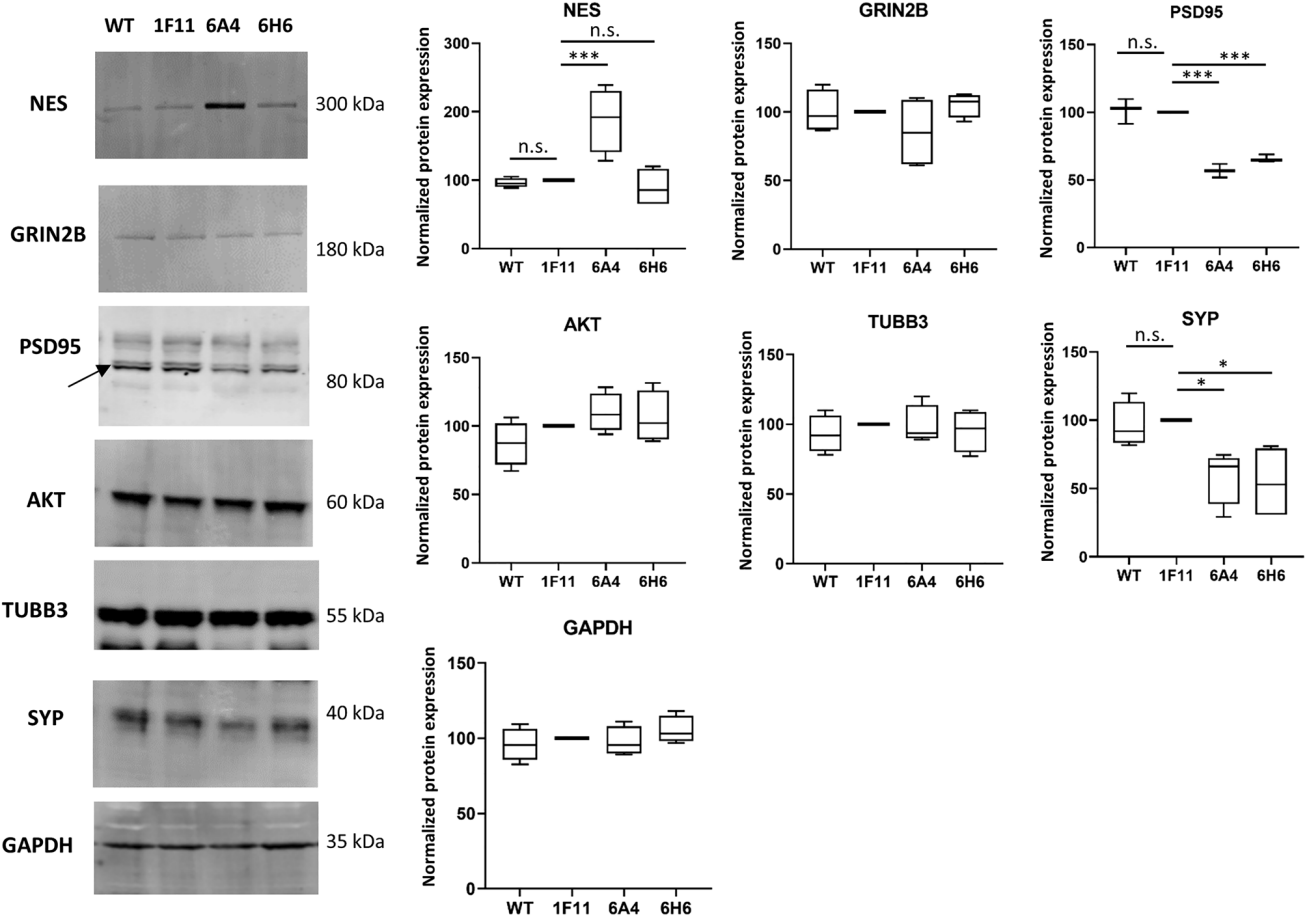
Quantification of protein expression in SH-SY5Y cells (7 days after differentiation). Western blot analysis with protein from two control and two *SHANK2* mutant cell lines. The protein expression levels of NESTIN (NES), GRIN2B, PSD95, AKT, β3-Tubulin (TUBB3) and SYP were normalized against the expression of GAPDH (reference). GAPDH expression was analyzed against total protein expression and a differential expression of GAPDH between the different cell lines was ruled out. No differences between the two control lines WT and 1F11 could be detected. Differential expression levels between mutant and control cell lines were found for NES, PSD95 and SYP. Cropped western blot membrane images are shown and full-length blots are presented in Supplementary Fig. 8. n = 5 experiments, one-way ANOVA was carried out and protein expression of the two mutant cell lines (6A4, 6H6) and the wildtype cell line (WT) was compared against the control 1F11 (ANOVA results: NES F = 11.57, P = 0.0007; GRIN2B F = 1.21, P = 0.348; PSD95 F = 63,51, P < 0.0001; AKT F = 1.91, P = 0.181; TUBB3 F = 0.28, P = 0.837; SYP F = 6.48, P = 0.0074; GAPDH F = 0.93, P = 0.458). n.s.—not significant. Correction for multiple testing with Dunnett´s test (n = 3 tests for each protein). Data were presented as box-plots and the corrected P-values are indicated, *P ≤ 0.05, ***P ≤ 0.001.

Next, we assessed the expression of several proteins by Western blot to elucidate the consequences of the loss of *SHANK2* regarding neuronal differentiation. SHANK2 expression was reduced in the undifferentiated state and after 7 days of differentiation in both *SHANK2* mutant lines (6A4, 6H6) compared to the control 1F11 (Fig. 1). We included the non-edited wildtype SH-SY5Y cells (WT) in the Western blot analysis as a second control cell line, and compared expression levels to 1F11. The comparison between the two control lines revealed similar expression levels for WT and 1F11 for all analyzed proteins (Fig. 4). A comparable expression of all tested genes was also shown on mRNA level (Supplementary Fig. 7).

**Figure 5:**
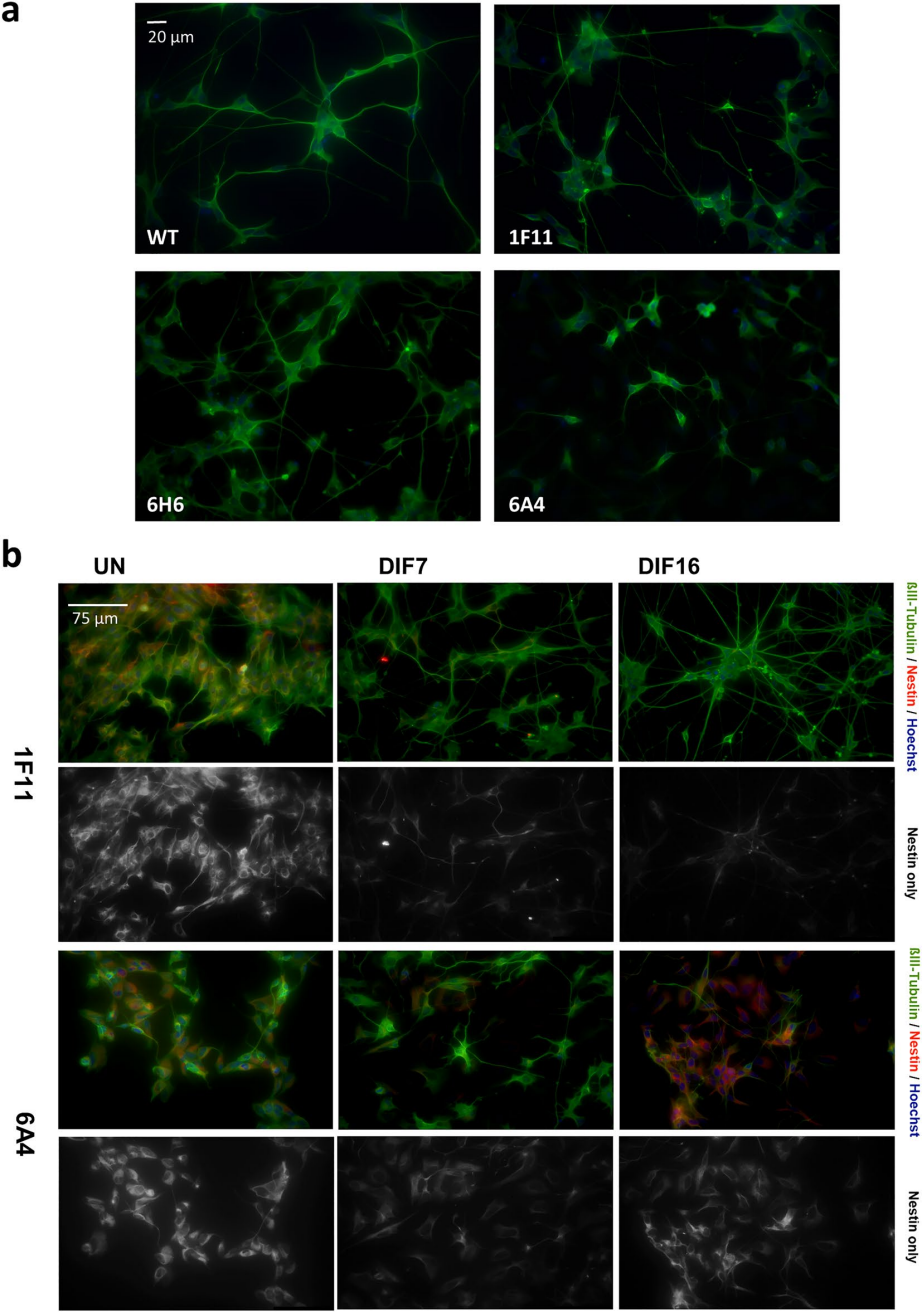
Immunofluorescence microscopy of *SHANK2* mutant cells. (**a**) β3-Tubulin expression after 7 days of differentiation (DIF7) illustrating the morphology of control and *SHANK2* mutant cell lines. (**b**) Nestin and β3-Tubulin co-staining using immunofluorescence microscopy. During 16 days of neuronal differentiation, the number of cells showing Nestin expression decreased in the control line (1F11), and nearly all cells developed neuronal morphology. In comparison, cells expressing Nestin could be detected throughout the differentiation period in the genome edited cell line (6A4). UN undifferentiated, DIF7 7 days of differentiation, DIF16 16 days of differentiation.

To elucidate the influence of *SHANK2* mutations on differentiated cells, we compared protein expression levels between the control 1F11 and the two mutant cell lines after 7 days of differentiation. The heterozygous *SHANK2* mutant line 6H6 showed significantly reduced amounts of the pre- and postsynaptic proteins SYP and PSD-95, whereas other proteins were not affected (Fig. 4, Supplementary Fig. 8). The compound heterozygous *SHANK2* mutant line 6A4 had highly increased Nestin levels and significantly reduced levels of SYP and PSD-95, indicating impaired neuronal differentiation (Fig. 4, Supplementary Fig. 8). No differences in protein expression could be detected for β3-Tubulin, GRIN2B and AKT (Fig. 4, Supplementary Fig. 8). With β3-Tubulin immunocytofluorescence microscopy staining, we detected long dendritic protrusions starting to form networks after 7 days of differentiation in the controls (WT, 1F11) and the heterozygous *SHANK2*-mutant cell line 6H6 (Fig. 5a). The compound heterozygous *SHANK2* cell line 6A4 lost this ability, showing less and shorter dendritic protrusions (Fig. 5a). As Nestin expression showed the most prominent change on transcriptome level in this cell line, we further investigated its expression by immunofluorescence microscopy and performed a co-staining of Nestin and β3-Tubulin during neuronal differentiation (Fig. 5b). We compared cell line 6A4 to the control 1F11 in the undifferentiated stage and after 7 and 16 days of differentiation. Both cell lines started to differentiate into neurons and Nestin-positive cells disappeared in the control cell line 1F11, whereas Nestin-positive cells could still be detected throughout differentiation in cell line 6A4. The Nestin-positive cells showed a different morphology with less cellular protrusions compared to β3-Tubulin-positive cells, even at an advanced stage of differentiation (Fig. 5b).

### *SHANK2* mutations affect tyrosine kinase receptor downstream signaling pathways

**Figure 6:**
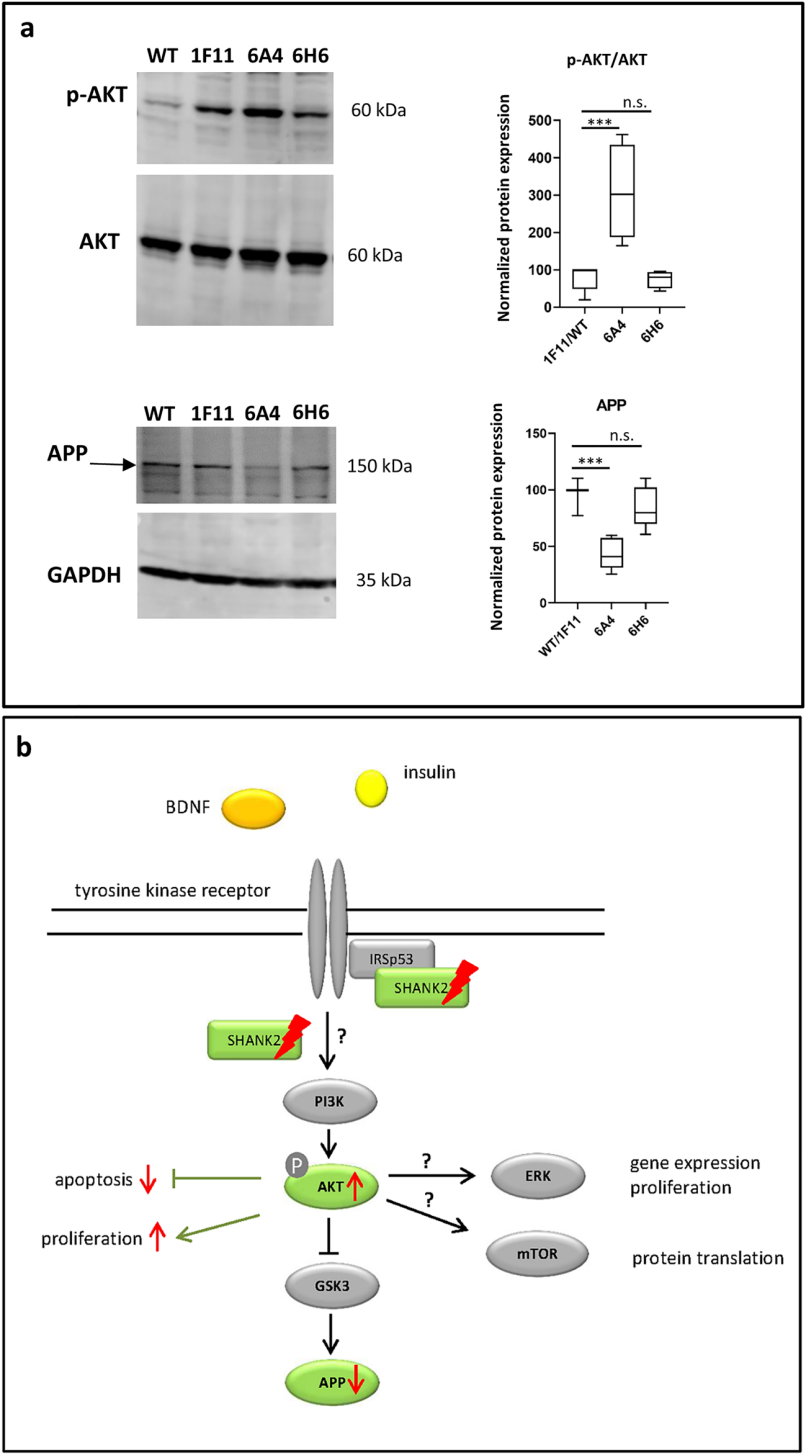
Analysis of the influence of *SHANK2* mutations on AKT serine/threonine kinase (AKT) and amyloid precursor protein (APP) expression (7 days of differentiation) (**a**) and Overview of the putative influence of *SHANK2* mutations on tyrosine kinase receptor downstream signaling (**b**). (**a**) Western blot analysis was carried out to identify p-AKT levels, as an indicator for tyrosine kinase receptor signaling pathway activity, and APP expression levels. P-AKT and AKT levels were normalized against GAPDH and the ratio between the normalized p-AKT/AKT expression calculated. APP expression was normalized against GAPDH. The mean for both controls (WT and 1F11) was taken for comparison with the *SHANK2* mutant lines. The cell line with compound heterozygous *SHANK2* mutation (6A4) showed impaired p-AKT levels and APP expression levels. Cropped western blot membrane images are shown and full-length blots are presented in Supplementary Fig. 9. n = 5 experiments, one-way ANOVA (ANOVA results: p-AKT F = 16.65, P = 0.0003; APP F = 25.83, P < 0.0001). Each mutant line was compared against the mean of both controls. ***P ≤ 0.001, correction for multiple testing with Dunnett’s test. Data were presented as box-plots. (**b**) Scheme illustrating the potential regulation of AKT signaling in the differentiating SH-SY5Y cells downstream of BDNF/TRKB or insulin/insulin receptor signaling. Increased p-AKT levels probably led to reduced apoptosis, increased proliferation and reduced amounts of amyloid precursor protein (APP). Further investigation is needed regarding the ERK and mTOR signaling downstream of AKT, to elucidate an effect from *SHANK2* mutations. These pathways have been already linked to ASD pathology^61^. The part of the signaling cascade which was analyzed in this study is illustrated in green. In this simplified scheme not all different factors which influence AKT and APP expression can be considered. *BDNF* brain derived neurotropic factor, *TRKB* tyrosine receptor kinase beta, *ERK* extracellular-signal regulated kinase, *mTOR* mammalian target of rapamycin.

It has been hypothesized that insulin signaling contributes to the development of autism in genetically susceptible individuals^15^. As SHANK2 (as well as SHANK1 and SHANK3) directly interacts with IRSp53 (insulin receptor substrate p53), it may also play a role in insulin signaling in the brain^17,37^. The serine/threonine kinase AKT acts downstream of the tyrosine kinase receptor signaling pathways. Therefore, we analyzed the phosphorylation levels of AKT in relation to overall AKT levels by Western blot analysis to get an insight into a putative impairment of insulin or, more generally, into tyrosine kinase receptor downstream signaling during early neuronal differentiation. Interestingly, the cells with bi-allelic *SHANK2* mutation showed an increase of phosphorylated AKT, (Fig. 6a, Supplementary Fig. 9), suggesting that a loss of *SHANK2* impairs downstream signaling of tyrosine kinase receptors such as insulin receptors during early neuronal differentiation.

### Bi-allelic *SHANK2* mutations influence amyloid precursor protein expression

APP regulates both synapse formation and neuronal plasticity and is expressed during neuronal differentiation^23–25^. Mutations in APP have been linked to AD^22,23^, in which SHANK expression was also found to be altered^11^. SHANK2 and APP function might be linked, as they are both implicated in synaptic signal transduction, and might interact during the course of neurodegenerative disorders. Therefore, we asked if *SHANK2* mutations can influence APP expression. The quantification of APP protein expression by Western blot analysis revealed a significant decrease of APP levels in the compound heterozygous *SHANK*2 mutant line, whereas cells with heterozygous *SHANK2* mutation showed no significant difference compared to controls (Fig. 6a, Supplementary Fig. 9).

## Discussion

In this study, the molecular and cellular consequences of mono-allelic and bi-allelic *SHANK2* frameshift mutations were investigated in the neuroblastoma cell line SH-SY5Y. The mutations were introduced by CRISPR/Cas9 genome editing in exon 23—encoding for part of the proline-rich region—in proximity to a previously identified nonsense mutation from a patient with ASD (R841X)^4^. Deleterious de novo* SHANK2* mutations have always been identified in a heterozygous state in patients with ASD, intellectual disability, attention deficit and hyperactivity disorder as well as language impairment, with one allele unaffected^2–5^. Therefore, a heterozygous *SHANK2* mutant line is the appropriate model to identify impairments that contribute to the pathology in patients. Nevertheless, the compound heterozygous line is useful to explore *SHANK2* function in human neurons. Our data showed that both mono- and bi-allelic mutations of *SHANK2* led to reduced wildtype SHANK2 protein levels in SH-SY5Y cells. The bi-allelic mutation of *SHANK2* in SH-SY5Y cells initially resulted in strongly reduced *SHANK2* mRNA levels compared to the control in the undifferentiated state which is in line with reduced protein levels. After 7 days of differentiation *SHANK2* mRNA levels were equal between the cell lines even though protein levels were strongly reduced and after 16 days of differentiation even an increase of *SHANK2* mRNA expression was found for the compound heterozygous cell line (Supplementary Fig. 4). This might be caused by a compensatory mechanism taking place during neuronal differentiation leading to an upregulation of gene transcription because the protein is missing. The treatment of the cells during differentiation with retinoic acid and BDNF might also stimulate *SHANK2* transcription.

The parallel analysis of both the compound heterozygous and the heterozygous lines revealed shared and distinct alterations in cell function. Similar consequences in both cell lines were identified concerning lower apoptosis rates, and reduced pre- and postsynaptic protein levels.

At the beginning of neuronal differentiation, cells have to switch from proliferation to differentiation^38^. Both *SHANK2* mutant lines had lower apoptosis rates after 7 days of differentiation, and the compound heterozygous line also showed higher proliferation compared to controls. This may point to a role of SHANK2 regarding the switch from proliferation to differentiation during the early neuronal differentiation process in neuroblastoma cells. Both cell lines showed diminished pre- (SYP) and postsynaptic (PSD95) protein expression after 7 days of differentiation. SHANK2 is known to interact with PSD95 through the guanylate kinase-associated protein (GKAP) to anchor the AMPA and NMDA receptors at the postsynaptic density^39^. Glutamatergic markers were affected by *SHANK2* mutations with reduced PSD95 and *GRIA2* expression. So far, the role of SHANK2 has been mainly investigated in mature synapses^30^, whereas our results suggest that it may already have important functions during early neuronal differentiation, even prior to synapse formation. Interestingly, despite being a predominantly postsynaptic protein, the *SHANK2* mutations also affect presynaptic protein expression, as shown by diminished SYP expression.

In the cell line with bi-allelic *SHANK2* mutations expression levels of other autism-related genes and proteins (according to the SFARI Gene database^6^) were affected, such as *SHANK1, SHANK3, GABRB3, GRIA2* and PSD95. This may point to synergistic effects and also emphasizes the importance of these genes and proteins—as well as the signaling cascades they belong to—in the pathology of autism. Cells with bi-allelic *SHANK2* mutations remained in a neuronal precursor cell state, as shown by high Nestin levels and impaired neurite outgrowth. They lost their ability to extend long neurite protrusions and to form networks, which is in line with diminished levels of the pre- and postsynaptic proteins SYP and PSD95. All these observations indicate a severe impairment of neuronal differentiation. The strongest effects of Shank2 deletions were only seen in the cell line with bi-allelic SHANK2 mutation, which compares to the rat and mouse models that show the strongest phenotype only when both *Shank2* alleles are deleted^27–29^. In humans bi-allelic mutations have not yet been described and may lead to embryonic death. Human individuals with heterozygous deleterious de novo* SHANK2* mutations show a general developmental delay with delayed development of speech as well as learning impairments and mild to severe intellectual disability^4,5^. This may be a long term consequence of impaired neuronal differentiation which cannot be fully compensated. In the homozygous *Shank2* knockout mice, cognitive impairments as well as a disturbed synaptic signal transmission have been observed^28,29^. The very early neuronal development in utero or during the first postnatal days has not been studied in these animals, therefore we do not know if an impaired neuronal differentiation may precede impaired synaptic signaling.

In a recent publication, the SHANK2-R841X mutation was investigated in human iPSC-derived cortical neurons^31^. The authors also showed that SHANK2-deficiency had an impact on dendritic growth, resulting in increased dendritic length and complexity, and led to a perturbation of neurodevelopmental gene set expression^31^. This is in line with some of our findings, even though the data between the two studies cannot be directly compared. The work on human cortical neurons (HCN2) by Luo et al.^40^ showed that reduced SHANK2 expression decreased neurite numbers and length in human neurons, which is comparable to our findings of reduced neurite outgrowth in the compound heterozygous *SHANK2* mutant line^40^. They also report an impact on proliferation and apoptosis, but in their study, low SHANK2 expression inhibited proliferation and promoted apoptosis, whereas increased proliferation and decreased apoptosis was found in our study. The differences between our work and those two studies can be explained by two facts: (a) the cell models used are notably different, despite the fact that all models are human cell lines; (b) the focus of our research was on the onset of neuronal differentiation, whereas Zaslavsky et al.^31^ and Luo et al.^40^ used differentiated cortical neurons.

The severe impairment of neuronal differentiation might be partly explained by an increase of tyrosine kinase receptor signal activity. Interestingly, 6A4 cells showed elevated p-AKT levels, indicating more active tyrosine kinase receptor downstream signaling. During the process of neuronal differentiation, cells were constantly treated with retinoic acid (RA) and brain-derived neurotropic factor (BDNF). RA is used to activate the transcription of gene sets to induce differentiation and inhibit cell proliferation^38^. BDNF binds to tyrosine receptor kinase beta and activates several signaling cascades including the phosphatidyl-inositol-3-kinase (PI3K)—AKT pathway, which, in turn, inhibits apoptosis, regulates protein synthesis and remodels the cytoskeleton^41^. In parallel, other growth factors like insulin are present in the culture medium, which activate further tyrosine kinase receptors like the insulin receptor. At synapses, the insulin receptor was found co-expressed with insulin receptor substrate p53 (IRSp53), which is phosphorylated upon stimulation with insulin and acts as key factor in cytoskeleton reorganization, hence mediating neurite outgrowth^42^. IRSp53 is a direct interaction partner of the SHANK proteins^16^, linking SHANK2 to insulin signaling. As we identified increased p-AKT levels in the 6A4 cell line, which occurred together with reduced apoptosis rates and increased proliferation, we concluded that bi-allelic *SHANK2* mutations might affect BDNF and/or insulin downstream signaling during early neuronal differentiation (Fig. 6b, Table 2). This is in line with other findings that describe the dysregulation of AKT signaling, which is part of the PI3K-AKT-mTor cascade downstream of tyrosine kinase receptor signaling, as a cause of ASD and other neurodevelopmental disorders^43,44^.

**Table 2:** Summary of results comparing cell lines with mono-(*SHANK2*^−/+^*)* and bi-allelic (*SHANK2*^−/−^) *SHANK2* mutations against the controls.

Analysis	*SHANK2*^−/−^ 6A4	*P*-value	*SHANK2*^−/+^ 6H6	*P*-value
Apoptosis (DIF7)	↓ 81%	< 0.0001	↓ 44%	0.0004
Proliferation	↑ 100%	< 0.0001	–	0.49
Neurite length	↓ 44%	< 0.0001		0.362
Protein expression	SHANK2-UN ↓89%	< 0.0001	SHANK2-UN ↓46%	< 0.0001
	SHANK2-DIF7↓85%	< 0.0001	SHANK2-DIF7↓53%	< 0.0001
	NES ↑ 87%	0.0019	–	0.8966
	PSD95 ↓ 43%	< 0.0001	PSD95 ↓ 34%	< 0.0001
	SYP ↓ 41%	0.025	SYP ↓ 45%–SYP ↓	0.0134
	p-AKT ↑ 200%	0.0003	–	0.9987
	APP ↓ 56%	< 0.0001	–	0.1773

The *SHANK2*^*−/−*^ cell line 6A4 showed more extensive alterations in cellular properties; – = no change. The increase is indicated by ↑ and the amount in %, a decrease is indicated by ↓ and the amount in %. Corrected P-values are shown. UN—undifferentiated cells, DIF7—cells after 7 days of neuronal differentiation. NES, PSD95, SYP, p-AKT and APP were analyzed only in cells after 7 days of differentiation.

In addition, we could also show that *SHANK2* mutations have an impact on APP expression, a protein extensively studied in the context of AD, although its exact biological function is not yet well understood^23^. The reduced APP expression in cells with bi-allelic *SHANK2* mutations may also contribute to the diminished neurite outgrowth and some of the neuronal differentiation impairments. These results point to a functional link between APP and SHANK2 during neuronal development. Low APP expression occurred together with high p-AKT levels in the cell line 6A4. APP expression is known to be regulated by growth factors like insulin and BDNF which activate PI3K and AKT. AKT further phosphorylates glycogen synthase kinase 3 (GSK3) which acts as an inhibitor and results in less translation of APP protein^45^ (Fig. 6b).

There are functional aspects of APP which make it interesting to look at in neurodevelopmental disorders: APP regulates synapse formation, neurite outgrowth and neuronal plasticity. Furthermore, it is expressed during early neuronal differentiation at sites where SHANK2 is also expressed^23–25,46^. In addition, there is increasing evidence showing that molecular and cellular processes involved in neurodevelopment are also relevant to the pathology of neurodegenerative diseases^47,48^. One connection could be the pathological reactivation during neurodegeneration of genetic programs physiologically relevant during neurodevelopment, especially in Alzheimer’s disease^49^. There is also a genetic overlap between intellectual disability and cognitive aging^48^. Since SHANK2 is involved in fundamental molecular and cellular processes in neurons, it is plausible that its role might also be relevant in neurodegeneration.

The results of our study should be considered with prudence, as they were obtained in vitro in SH-SY5Y cells. As these neuroblastoma cells were tumor-derived, it is not possible to investigate all aspects of neuronal properties. The intrinsic biological variation of the neuroblastoma cell population is likely to mask low effects resulting from *SHANK2* mutations, especially in the heterozygous state. Our marker gene expression analysis revealed significant expression differences, mostly in the compound heterozygous *SHANK2* mutant line. The cell line with a heterozygous *SHANK2* mutation may still show small changes that could not be identified due to the given limitations of our experimental setup. Despite the fact that three independent experiments were carried out, this may not allow the detection of alterations with very low effect sizes.

p-AKT levels were investigated as a general indicator of tyrosine receptor downstream signaling. Additional work is necessary to further specify which tyrosine kinase receptor signaling pathway is mostly influenced (e.g. BDNF, insulin, IGF1), which downstream signaling path of PI3K-AKT is mainly altered (e.g. mTOR, GSK3beta, ERK), and how this affects both the transcriptome and proteome.

In summary, we have shown that mono- and bi-allelic *SHANK2* frameshift mutations resulted in an impairment of early neuronal differentiation in SH-SY5Y cells, characterized by changes in cell growth properties and reduced pre- and postsynaptic protein expression. We also provided the first link between *SHANK2* mutations and impaired receptor tyrosine kinase downstream signaling, and revealed an impact of *SHANK2* mutations on APP expression. Further investigation is warranted, preferably in human iPSC-derived neuronal cells from patients with *SHANK2* mutations, to follow-up these novel insights into SHANK2-dependent pathways.

## Methods

### Cell culture

Human neuroblastoma cells (SH-SY5Y) were obtained from the Leibniz Institute DSMZ-German Collection of Microorganisms and Cell Cultures. The cells were grown on 75 cm flasks in Dulbecco’s modified Eagle medium (DMEM, Thermo Fisher Scientific), supplemented with 15% fetal calf serum, 1% non-essential amino acids and 1% Penicillin–streptomycin at 37 °C in a humidified environment with 5% CO_2_. Cells were split at 80–90% confluency and 2 × 10^5^ cells were plated per well on a 6-well cell culture plate for RNA and protein isolation. Undifferentiated cells were harvested 3 days after seeding. Cells were differentiated according to the protocol published by Chiocchetti et al.^32^ and harvested after 7 or 16 days of differentiation, with medium changes 3 times per week. For neurite tracing and immunocytochemistry, 7.5 × 10^4^ cells were seeded per well on a 24-well plate on glass coverslips coated with collagen. The cells were tested against mycoplasma contamination with the Venor GeM Classic mycoplasma detection kit for conventional PCR (Minerva Biolabs) and all used cell lines were free of mycoplasma. Genome edited cells were used for experiments with passages ranging from 15 to 20.

### Genome editing

The SHANK2-gRNA was cloned with two primers: SHANK2_G2FCACCGTCGAGGGATGCCCAGAAACG and SHANK2_G2R AAACCGTTTCTGGGCATCCCTCGAC into the pSpCas9(BB)-2A-GFP vector (Addgene, 48138) using the restriction site BBsI. The plasmid was then introduced into the SH-SY5Y cells using lipofectamine (Invitrogen). Four days after transfection, GFP-positive living cells were sorted by fluorescence-activated cell sorting (FACS) as single cells into 96 well plates. The cell sorter used was a BD FACS Aria II SORP system. After 1–3 months, colonies could be split and analyzed for mutations. Medium changes were performed weekly.

### Quantitative real time PCR (qPCR)

Total RNA from SH-SY5Y cells was extracted with the RNAqueous—Micro Kit (Invitrogen) according to the manufacturer’s instructions. Reverse transcription was performed using the SuperScript VILO cDNA Synthesis Kit (Invitrogen). qPCR was conducted using the SYBR Green Lo-Rox Fast Mix (Bioline) and the ABI 7500 Fast Real-Time PCR system (Applied Biosystems). Each sample was analyzed in triplicates. Relative mRNA levels were assessed using the relative standard curve method by normalization to the mean of following reference RNAs: glyceraldehyde 3-phosphate dehydrogenase (GAPDH) mRNA, heat shock protein family D (HSP60) member1 (HSPD1) mRNA and hypoxanthine phosphoribosyltransferase 1 mRNA (HPRT1)^50^. The mean value and the standard error of the mean (SEM) of the relative expression values are presented. The sequences for the oligonucleotides used are given in Supplementary Table 2.

### Nonsense mediated mRNA decay

2 × 10^5^ cells were seeded per well on a 6-well plate and cultured for 24 h. Then, culturing medium was changed to either mock treatment (2 ml medium + 50 µl water) or cycloheximide (CHX) treatment (100 µg/ml, CHX was dissolved in water) and cultured for 6 h. Then, cells were harvested, RNA was isolated and *SHANK2* expression quantified with the primers *SHANK2*_F and SHANK2_R (details in Supplementary Table 2). Values of three independent experiments were presented as box-plots.

### Sequencing

DNA was isolated from SH-SY5Y cells with the Quick-DNA Miniprep kit (ZYMO), the region flanking the *SHANK2* mutation site was amplified with primers SH2_RXCheck_F agctgcagcgaataggaaag and SH2_RXCheck_R tgggcttcaagatgacagaa with Hotstar Taq (Qiagen) and sent for Sanger sequencing (GENEWIZ, Leipzig, Germany).

### Immunofluorescence microscopy

Immunocytofluorescence analysis was performed on fixed SH-SY5Y cells, treated with 4% paraformaldehyde solution in PBS for 15 min at room temperature, using the primary antibodies anti-β3-Tubulin^51^ (Abcam, ab18207, knockout validated, 1:500 dilution), anti-Nestin (Abcam, ab22035, 1:100 dilution) or anti-Ki67^52^ (Abcam, ab16667, knockout validated, 1:500 dilution), and as secondary antibodies Alexa Fluor 488 goat anti-rabbit, Alexa Fluor 488 donkey anti-rabbit, Alexa Fluor 546 rabbit anti-mouse or Alexa Fluor 546 goat anti-mouse (Thermo Fisher Scientific, 1:1000 dilution). Nuclei were stained with Hoechst dye (Hoechst 33,342). The TUNEL assay was carried out with the in situ Cell Death Detection kit, Fluorescein (Roche) according to the manufacturer’s instructions.

For the analysis of neurite length, cells were fixed after 4 days of differentiation. Images were taken in the channel corresponding to the β3-Tubulin signal and neurites were traced using the “Simple neurite tracer” tool of the FiJi software^36^ to determine their length. Three independent experiments were carried out and cells from six images were analyzed for each cell line per experiment.

Ki67 and TUNEL signals were quantified by using the FiJi^36^ software and by normalizing the number of signals to the total number of nuclei (as determined by Hoechst 33342 staining). Three independent experiments were carried out and cells of six images were analyzed for each cell line per experiment.

For the follow-up of Nestin expression levels, three independent experiments were performed, and 6 representative images were selected per experiment. All coverslips were mounted using Aqua-Poly/Mount medium (Polysciences).

### Protein analysis

Protein extraction from SH-SY5Y cells was performed at 4 °C using RIPA buffer supplemented with SIGMAFAST protease inhibitor (S8820; Sigma). Protein concentrations were determined with the BCA protein assay kit (Pierce, Thermo Scientific). Western blot analysis was executed using the Odyssey Infrared Imaging System (LI-COR Biosciences). Twenty-five micrograms of proteins were separated on Novex WedgeWell 4–12% Tris Glycine Gels (Thermo Fisher Scientific) and transferred to PVDF membranes (Millipore). In a pilot experiment, protein concentrations from 15 to 40 µg were loaded which revealed that the amount of 25 µg protein per sample is in a linear quantification range for all analyzed proteins (Supplementary Figs. 10 and 11). PVDF membranes (Millipore) were probed with rabbit polyclonal β3-Tubulin^53^ (1:20,000; Abcam, ab18207, knockout validated), sheep anti-SHANK2^31^ (1:2,500; R&D Systems, AF7035), rabbit anti-GRIN2B^54^ (1:1,000; Abcam, ab65783), mouse anti-PSD95^55,56^ (1:500; NeuroMab, 75–028, knockout validated), rabbit anti-Synaptophysin^57^ (1:7,500; Abcam, ab32127, knockout validated), mouse anti-GAPDH^58^ (1:10,000; Novus Biologicals, NB300-328), mouse anti-Nestin^59^ (1:2,500; Abcam, ab22035), rabbit anti-AKT^45^ (1:1,000; Cell Signaling Technology, 9272), mouse anti p-AKT(Ser473)^45,60^ (1:1,000; Cell Signaling Technology, 4051) and rabbit anti-APP^45^ (1:500, Abcam, ab126732, knockout validated).

The secondary antibodies used were IRDye 800CW donkey anti-mouse, IRDye 680LT donkey anti-mouse, IRDye 680RD donkey anti-rabbit, IRDye 800CW donkey anti-rabbit or IRDye 800CW donkey anti-goat (1:15,000 dilution; LI-COR Biosciences). Immuno-positive signals were quantified using the Image Studio Lite 3.1 software (LI-COR Biosciences). The Page Ruler Prestained Protein ladder (10–180 kDa) and the Spectra Multicolor High Range Protein ladder (40–300 kDa; both Thermo Fisher Scientific) were used as protein size markers.

Protein expression was normalized to the amount of GAPDH and the values obtained for the reference line 1F11 were set to 100. P-AKT and AKT levels were first normalized against GAPDH and then the ratio of the normalized data P-AKT/AKT was calculated. The normalized values were presented in box-plots.

### Equipment and settings

Westernblot images in Figs. 1, 4 and 6 were acquired with the Image Studio Software 4.0 (LICOR) with the scanning intensity of 5.0 for channel 700 and of 4.5 for channel 800.

Immunofluorescence images in Fig. 2 were taken with the Leica DMI4000B microscope with the camera Leica-DFC3000G-0001062113 and the objective N PLAN L 20×/0.35 DRY using the Leica Application Suite X software in the red, green and blue channel. The acquisition was performed with the time and space resolution XY = 0.959 µm and Z = 4.490 µm, 12 bit images were taken with a resolution of 1296 × 966 pixels. All pictures were taken with the same settings. In the illustrated TUNEL and Ki67 pictures brightness was increased by 30% for all conditions in the Power Point software. Images in Fig. 3 were taken with the same microscope and the same settings only for the green channel, brightness was increased by 20% for all conditions in Power Point software.

Images in Fig. 5 were taken with the same microscope and camera and the objective HC PL APO 40×/0.85 DRY. The acquisition was performed with the time and space resolution XY = 0.395 µm and Z = 0.761 µm, 12 bit images were taken with a resolution of 1296 × 966 pixel. All pictures were taken with the same settings. Colour saturation was increased to 200% for all conditions in Power Point software.

### Statistical analysis

Prism 8 (GraphPad Software) software was used for data analysis. The Shapiro Wilk test was performed to test for normal distribution when sample sizes were ≥ 5. In the TUNEL assay, Ki67 quantification, analysis of neurite length and Western blot experiments, a one-way ANOVA test was used for the comparison of each *SHANK2* mutant line (6A4, 6H6) against the reference 1F11. In addition, the WT line was compared to 1F11. A P-value of ≤ 0.05 was considered nominally significant. The Dunnett’s test was used to correct for multiple testing (n = 3 tests). A two-tailed unpaired Student´s T-Test was performed for the pairwise comparison of protein expression in undifferentiated versus the 7 days differentiated WT cell line.

Two-way ANOVA was performed for the comparison of RNA expression levels between the reference 1F11 and the two mutant lines (6A4, 6H6), with gene and cell line as the two factors; the Dunnett’s test was used to correct for multiple testing for each time point.

## Supplementary Information

Supplementary Information.

## Data Availability

The datasets supporting the conclusions of this article are included within the article and its additional files.
