# Supplementary material for: SHANK2 mutations impair apoptosis, proliferation and neurite outgrowth during early neuronal differentiation in SH-SY5Y cells
Source: Sci Rep. 2021 Jan 22;11:2128. doi: 10.1038/s41598-021-81241-4 (PMC7822837; doi:10.1038/s41598-021-81241-4)
Supplement: Supplementary file 1 — Supplementary Information. [file 41598_2021_81241_MOESM1_ESM.pdf]

Supplementary Information

SHANK2 mutations impair apoptosis, proliferation and neurite outgrowth during early neuronal differentiation in SH-SY5Y cells

Christine Unsicker[1], Flavia-Bianca Cristian[1], Manja von Hahn[1], Volker Eckstein[2], Gudrun A. Rappold[1], Simone Berkel[1*]

1) Department of Human Molecular Genetics, Institute of Human Genetics, University Hospital Heidelberg, Germany

2) Department of Internal Medicine V, University Hospital Heidelberg, Heidelberg, Germany

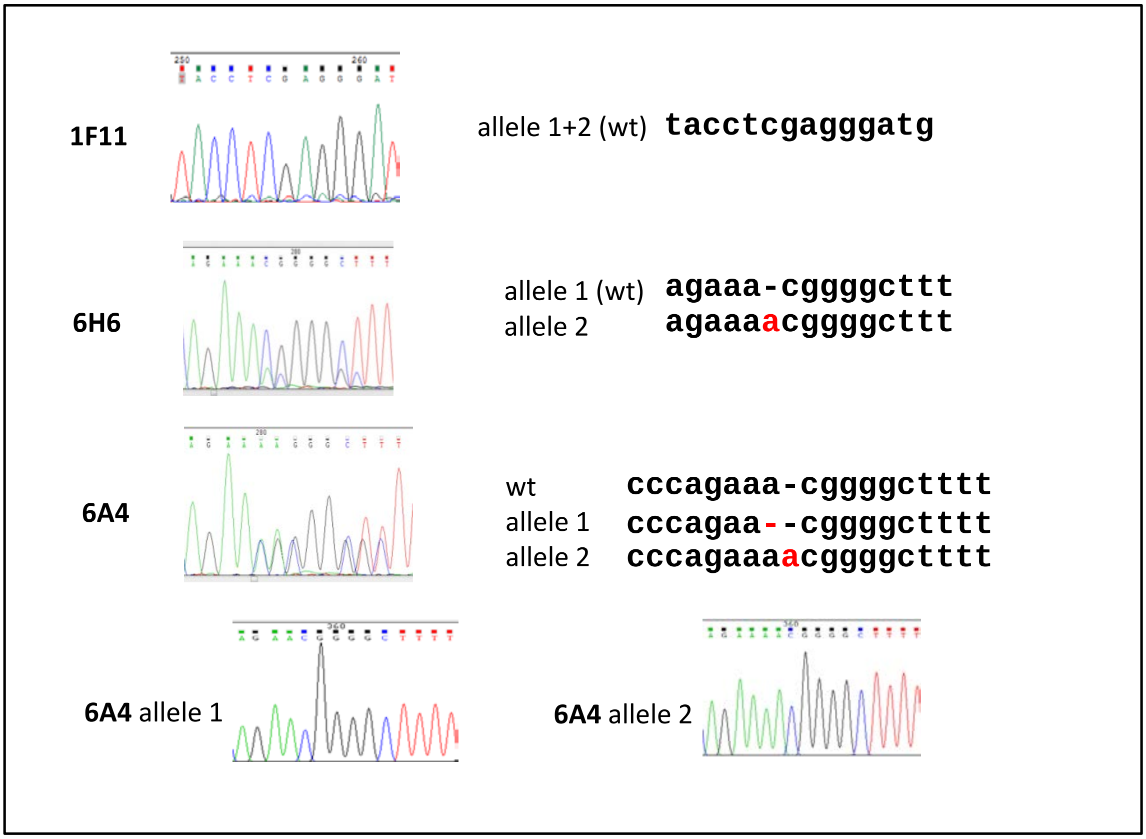

Supplementary Figure 1 Confirmation of SHANK2 mutation by Sanger Sequencing.

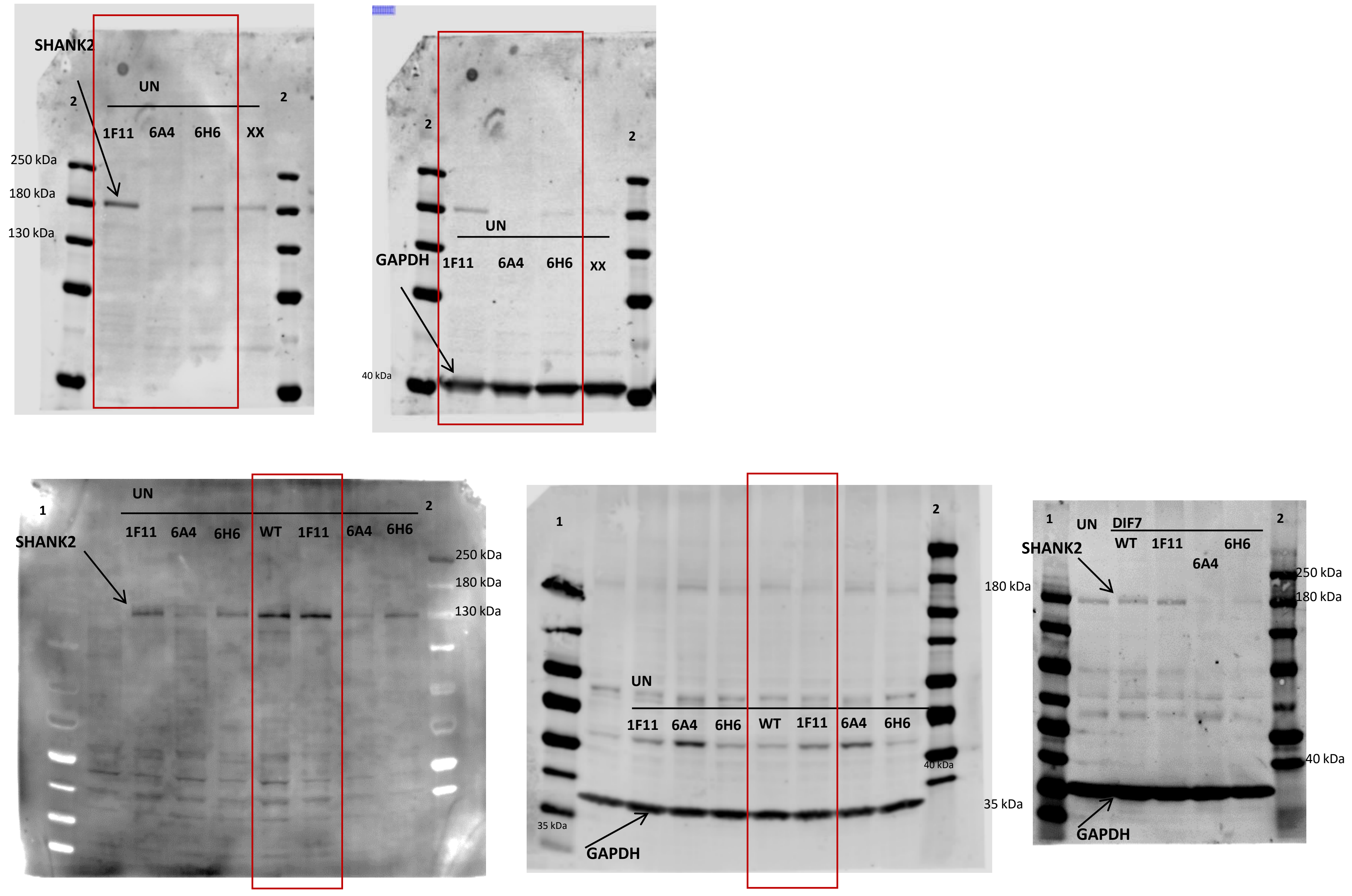

Supplementary Figure 2. Uncropped Western blot pictures are shown linked to Figure 1 – SHANK2 expression in genome-edited cell lines
1 Page Ruler pestained protein ladder (10-180 kDa)
2 Spectra Multicolor High Range Protein ladder (40-300 kDa)
UN- undiffentiated, DIF7- 7 days of differentiation, XX – cell line not used in this study

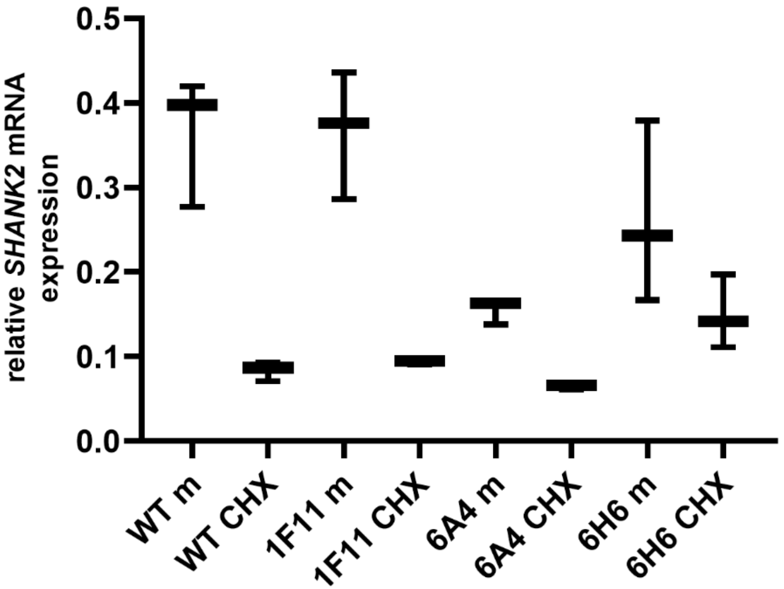

Supplentary Figure 3 Analysis of nonsense-mediated mRNA decay Quantification of *SHANK2* mRNA expression by qPCR (normalized against the reference genes GAPDH, HSPD1, HPRT1) revealed no evidence for nonsense – mediated mRNA decay in undifferentiated SHANK2-mutant cells after treatment with Cycloheximid (CHX) or mock (m). n=3 experiments

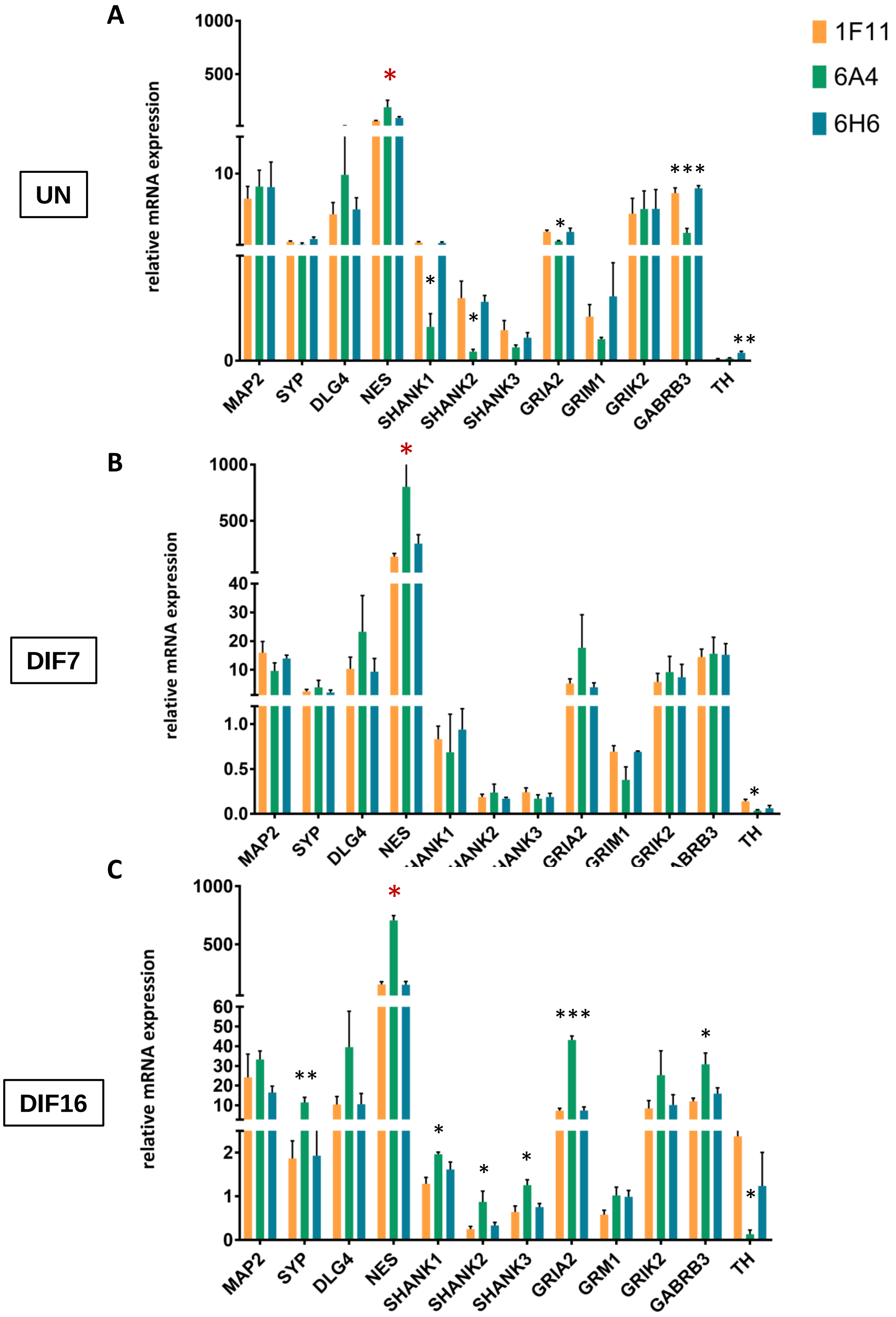

Supplementary Figure 4 Gene expression analysis of different marker genes. Quantification of marker gene mRNA expression by qPCR (normalized against references GAPDH, HSPD1, HPRT1). * P ≤ 0.0001 after correction for multiple testing using Dunnett´s test, two-way ANOVA with gene and cell line as factors. Comparison of mutant against the control (1F11) line for each gene revealed nominal significance for some markers, one-way ANOVA * P≤ 0.05, ** P≤ 0.01, *** P≤ 0.001, **A**) undifferentiated (UN), **B**) 7 days of differentiation (DIF7, **C**) 16 days of differentiation (DIF16), n= 3 experiments

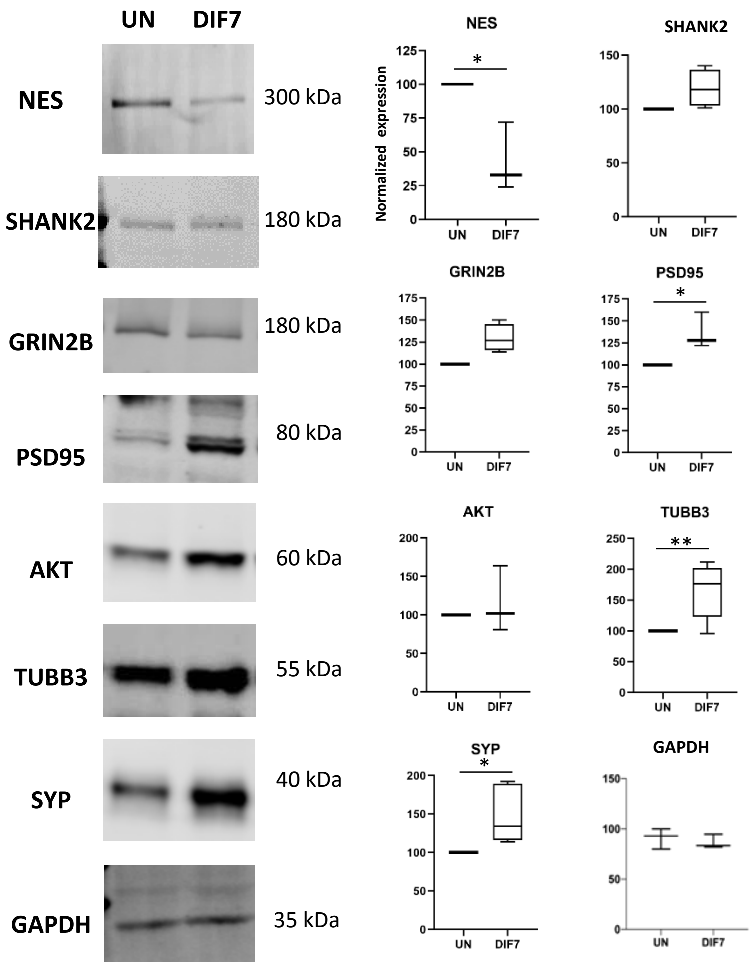

Supplementary Figure 5 Protein expression during neuronal differentation

Western blot analysis in the WT control cell line comparing expression levels between undifferentiated and 7 days differentiated cells. Cropped western blot membrane images are shown and full membrane pictures are shown in Supplementary Figure 6. Protein expression levels were normalized against the expression of GAPDH and the values of the undifferentiated cells was set to 100. GAPDH was quantified by normalization against the amount of total protein. Expression of PSD95, TUBB3 and SYP increased after 7 days of differentiation, whereas Nestin (NES) decreased. N= 5 experiments, two-tailed, unpaired Student´s T-test (NES P= 0.018, SHANK2 P= 0.0673, GRIN2B P= 0.1877, PSD95 P= 0.0359, AKT P= 0.563, TUBB3 P= 0.0099, SYP P= 0.0198, GAPDH P=0.673) * P≤ 0.05, ** P≤ 0.01.

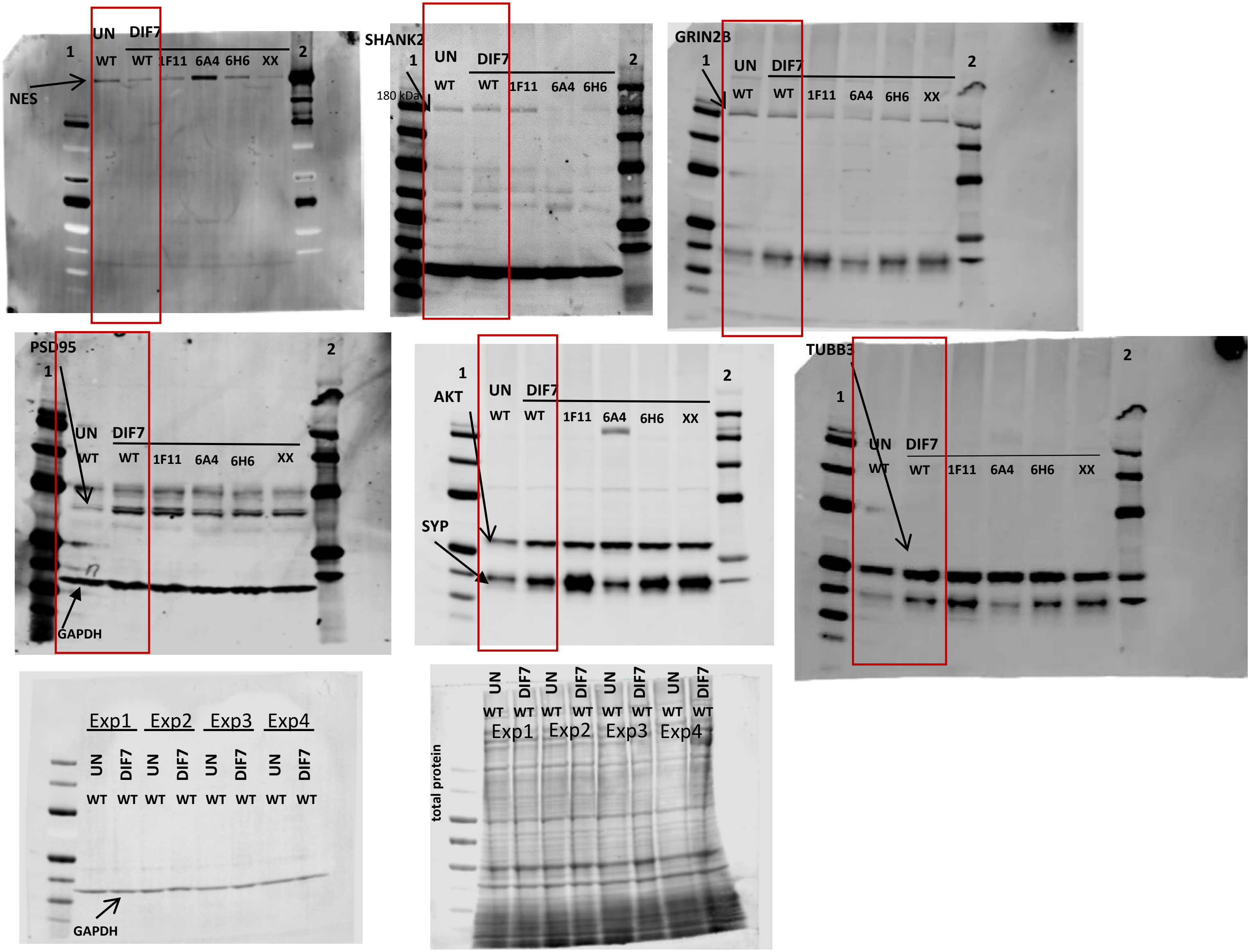

Supplementary Figure 6 Uncropped Western blot pictures linked to Supplementary Figure 5 - Protein expression during neuronal differentation . **1** Page Ruler prestained protein ladder (10-180 kDa), **2** Spectra Multicolor High Range Protein ladder (40-300 kDa), UN- undiffentiated, DIF7- 7 days of differentiation, XX – cell line not used in this study, Exp - experiment

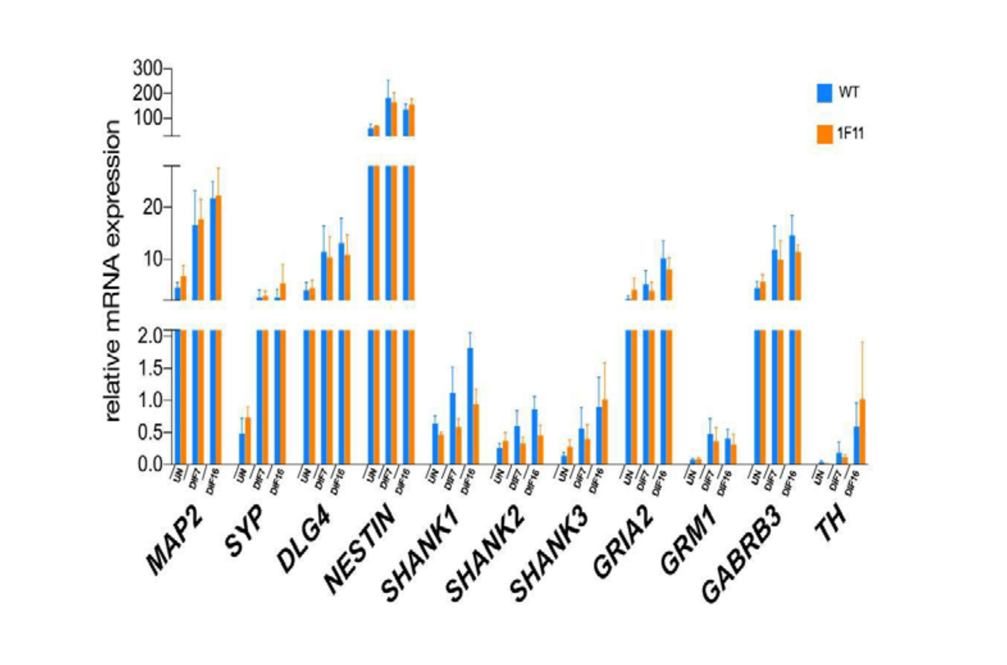

Supplementary Figure 7 Equal mRNA expression levels in control lines

No significant difference between expression levels for all markers on mRNA level could be detected using qPCR analysis. Expression was normalized against references GAPDH, HSPD1 and HPRT1. n=3 experiments, one-way ANOVA. All P-values exceeded the significance level of $P \leq 0.05$. All 11 genes were analyzed independently for each differentiation stage. UN – undifferentiated, DIF7- 7 days of differentiation, DIF16 – 16 days of differentiation

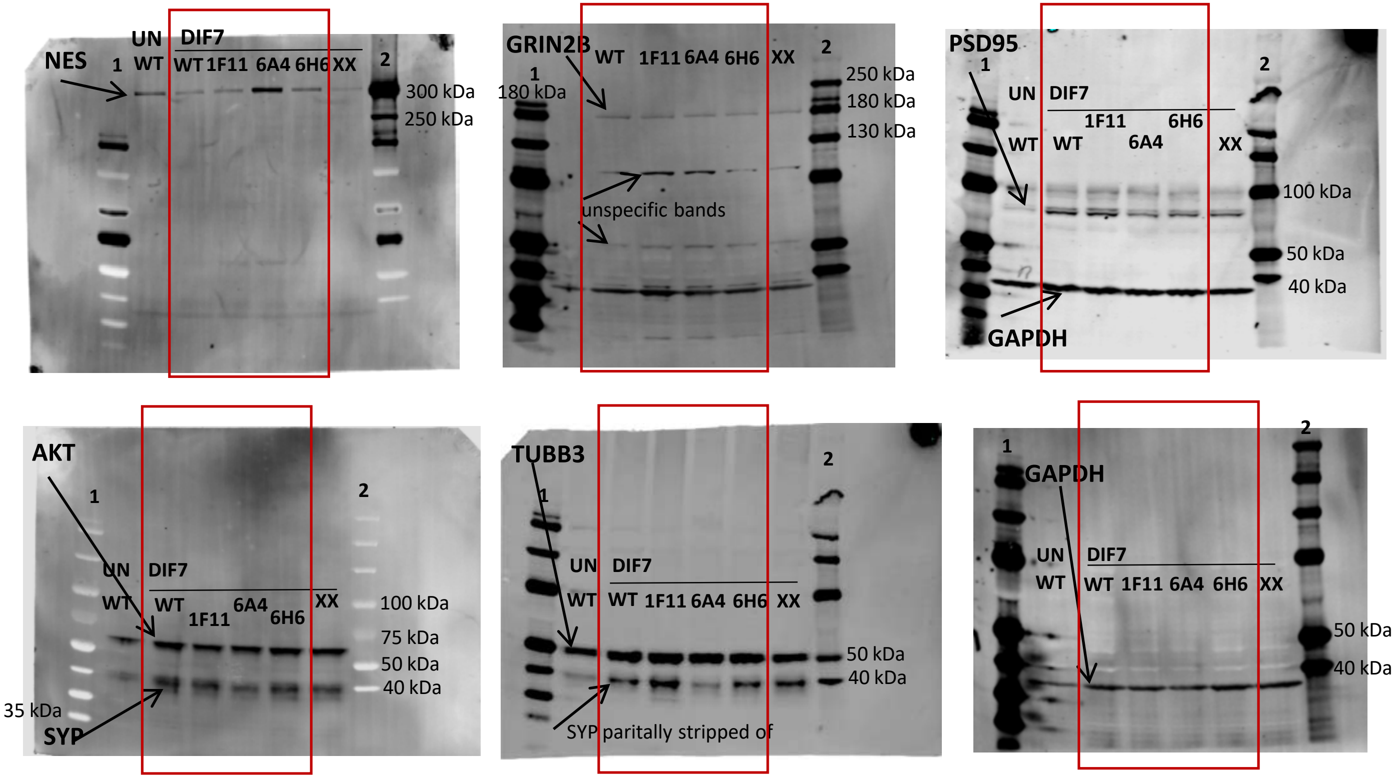

Supplementary Figure 8 Uncropped Western blot pictures are shown linked to Figure 4 – Quantification of protein expression in SH-SY5Y cells

1 Page Ruler pestained protein ladder (10-180 kDa)

2 Spectra Multicolor High Range Protein ladder (40-300 kDa)

XX – cell line not used in this study, DIF7 – 7 days of differentiation, UN - undifferentiated

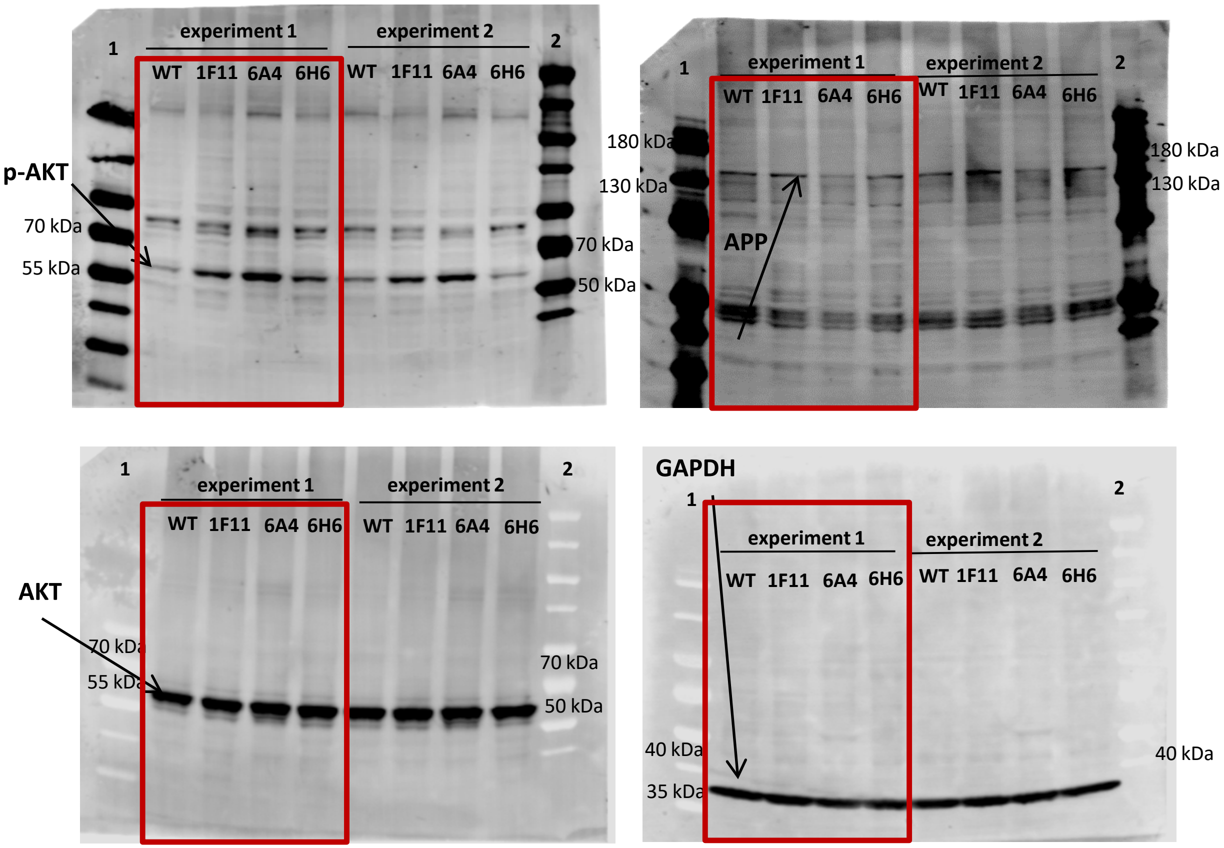

Supplementary Figure 9 Uncropped Western blot pictures are shown linked to Figure 6 – Analysis of p-AKT and APP expression in SH-SY5Y cells. **1** Page Ruler prestained protein ladder (10-180 kDa), **2** Spectra Multicolor High Range Protein ladder (40-300 kDa).).

a) undifferentiated WT SH-SY5Y cells

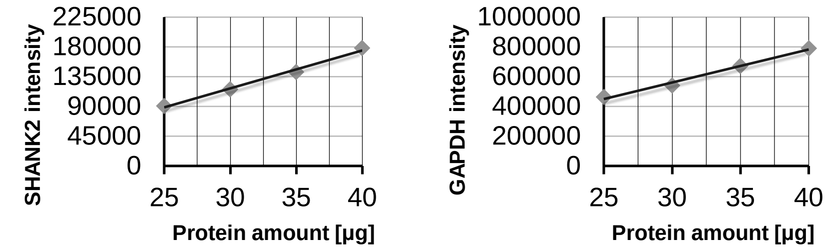

b) DIF7 WT SH-SY5Y cells

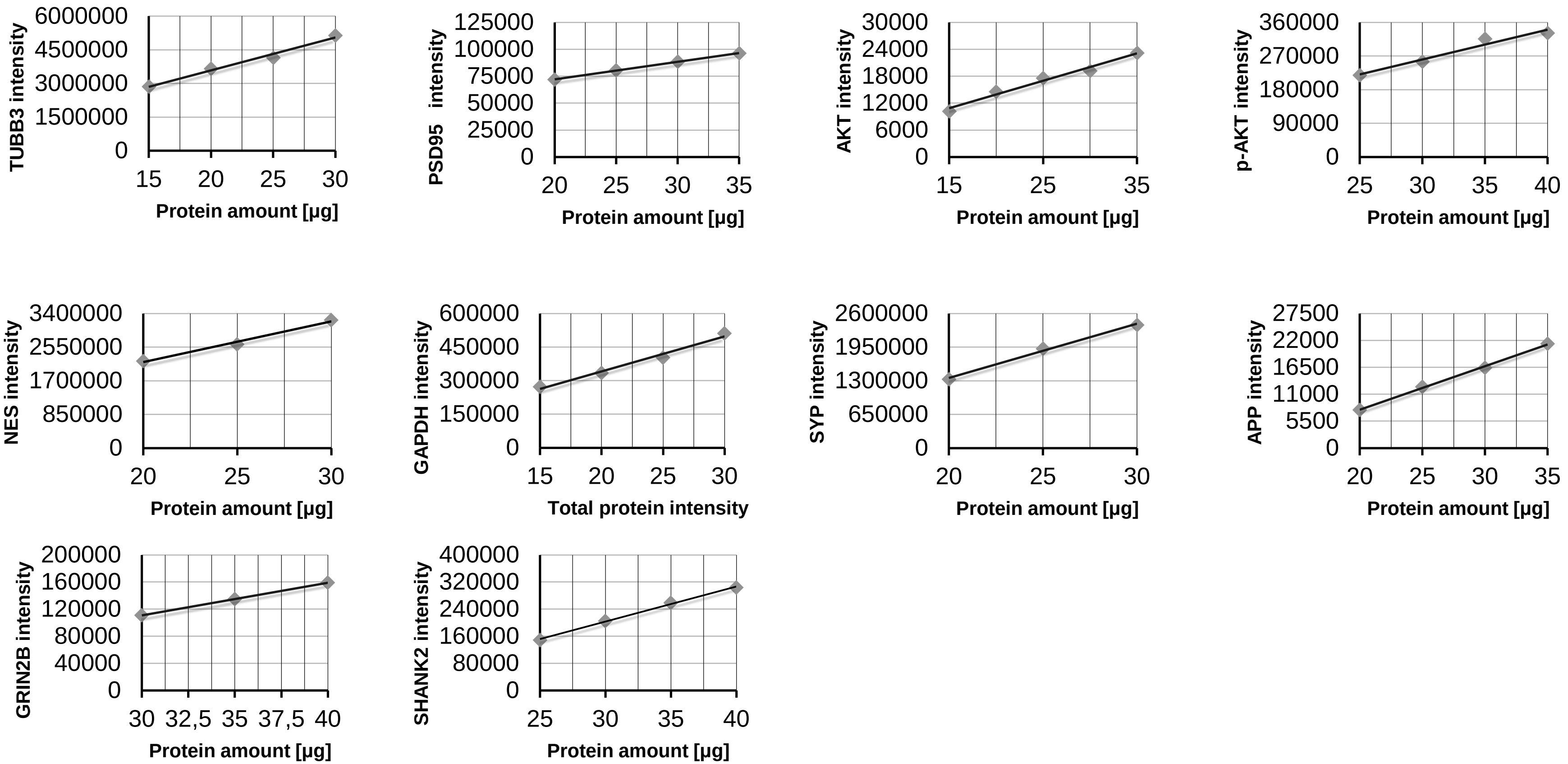

Supplementary Figure 10 Quantification of the loading controls. Using 25 µg of protein is in a linear quantification range for all analyzed proteins.

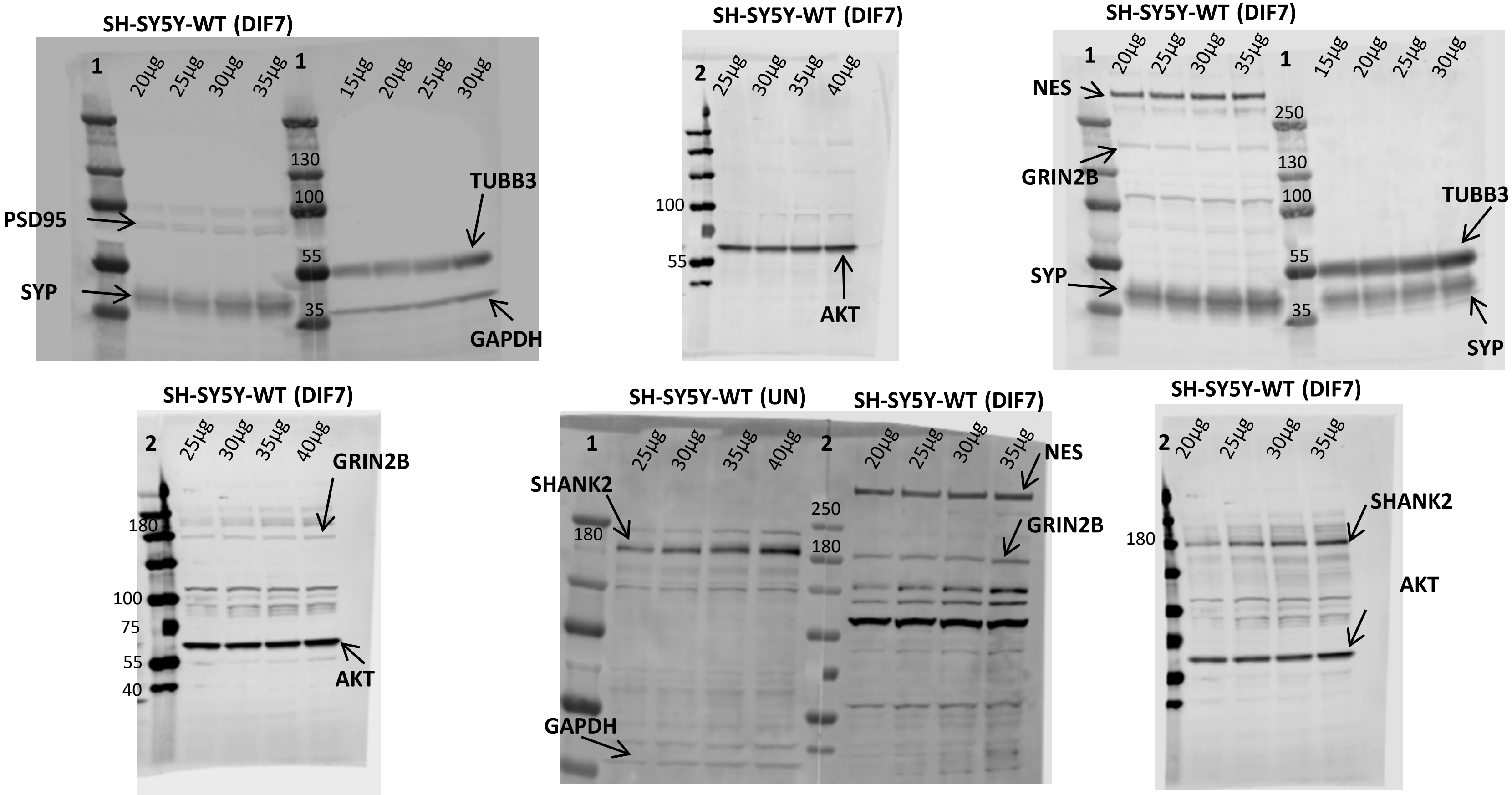

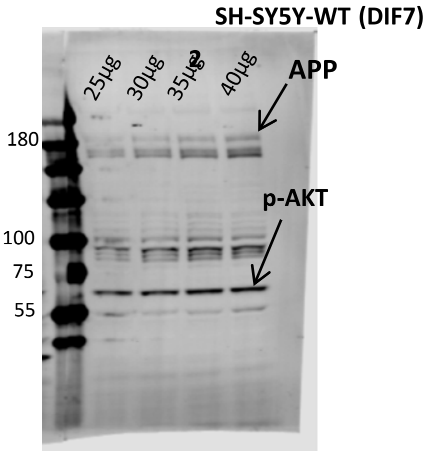

Supplementary Figure 11 Western blot membrane pictures linked to Supplementary Figure 10. UN- undifferentiated, DIF7- 7 days of differentiation, NPCs – neuronal precursor cells, **1** Page Ruler Plus prestained protein ladder (10-250 kDa), **2** Spectra Multicolor High Range Protein ladder (40-300 kDa)

Locus/Gene/Primer pair	Targeted sequence	Sequencing results
**Neuron navigator 2 isoform 3, (*NAV2*)**<br>**>gi|528476600:19678110-19678622**<br>NN_OT_F<br>AAGGCCTGCAGAGGGAAGT<br>NN_OT_R<br>ACCATCTGCCTCTGGATGAG	TTTCTGGGCATCC	WT<br>1F11<br>6A4<br>6H6<br>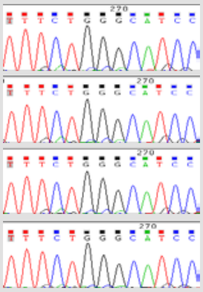
**PHD finger protein 21A isoform a (*PHF21A*)**<br>**>gi|528476600:45975059-45975571**<br>PHD_OT_F<br>TTCTCTCGTATTTCCCCCTTT<br>PHD_OT_R<br>GGAATTGATTTGCCCTTCCT	GGATGCCCAGAAA	WT<br>1F11<br>6A4<br>6H6<br>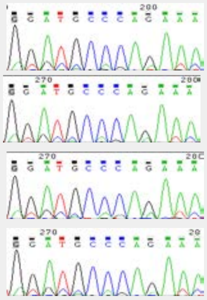
**RNA pseudouridylate synthase domain-containing protein 4 (*RPUSD4*)**<br>**>gi|528476600:125967576-125968100**<br>RDP4_OT_F<br>CCGCCTTCGACTAATTTGAG<br>RDP4_OT_R<br>TTGTAGTCGCCCTTCTCCAC	CCCGTTTCTGGGC	WT<br>1F11<br>6A4<br>6H6<br>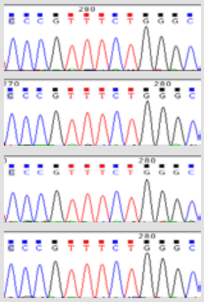
**glycogen phosphorylase, muscle form isoform 2 (*PYGM*)**<br>**>gi|528476600:64408302-64408915**<br>**GlyP_OT_F**<br>**AAGCAATCTGCTCACCTTGG**<br>**Glyp_OT_R**<br>**CAGAAAAGGGGAGGGAAGAG**	CCCCGTTTCTGGGC	WT<br>1F11<br>6A4<br>6H6<br>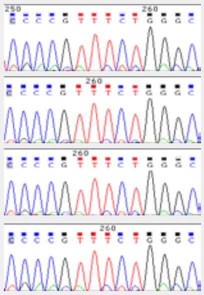
**potassium voltage-gated channel subfamily KQT member 1 isoform *(KCNQ1)***<br>**>gi|528476600:2601009-2601621**<br>K_OT_F<br>GACCCAGTGCTAGGAGGATG<br>K_OT_R<br>GAGTTGCAGAGGCAGACACA	CCCGTTTCTGGGC	WT<br>1F11<br>6A4<br>6H6<br>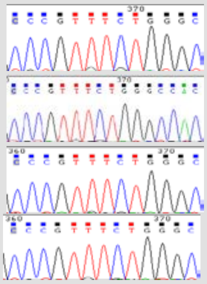

Gene/Locus/Primer pair	Targeted sequence	Sequencing results
**potassium voltage-gated channel subfamily C member 1 isoform *(KCNC1)*<br>>gi|528476600:17790832-17791444**<br>KC_OT_F<br>GGACAGATGGATGGAAGGAA<br>KC_OT_R<br>TGGACCTTCCAAACCACATT	CCCGTTTCTGGGC	WT<br>1F11<br>6A4<br>6H6<br>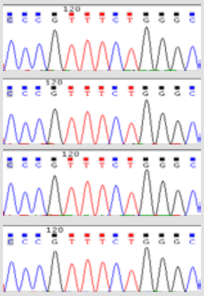
**thyrotropin releasing hormone *(TRH)*<br>>gi|485464565:1253-1890**<br>TRH_OT_F<br>TCCCTGACCATAAGCCTGAG<br>TRH_OT_R<br>TGATTATCTGGATGCAGGTTT	TTTCTGGGCATCCCT	WT<br>1F11<br>6A4<br>6H6<br>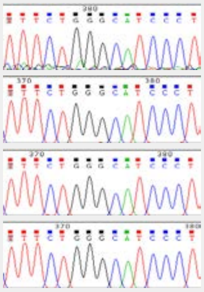
**cathepsin D preproprotein *(CTSD)*<br>>gi|528476600:1781977-1782590**<br>CatD_OT_F<br>GCGGGTACCTGTCACTCC<br>CatD_OT_R<br>AGAACTCAGGACCCCGATCT	GTTTCTGGGCATCC	WT<br>1F11<br>6A4<br>6H6<br>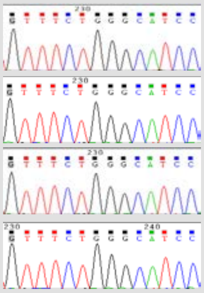
**open reading frame 203 (C20orf203) , chromosome 20<br>>gi|292658848:373-987**<br>Chr20_OT_F<br>CACATCAGTCTTGGCTGGAC<br>Chr20_OT_R<br>GTAACAGGGGACACCCTGAG	GAGGGATGCCCAGAA	WT<br>1F11<br>6A4<br>6H6<br>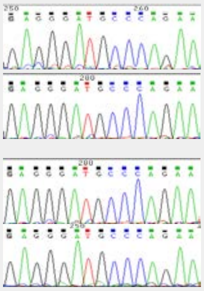
Synaptotagmin 9 (SYT9)<br>**>gi|528476600:7308850-7309362**<br>Syn9_OT_F<br>CCTGATGCTGCTCCTGTTTT<br>Syn9_OT_R<br>GGTCCCTAGATAGGATCTTTGC	GGATGCCCAGAAA	WT<br>1F11<br>6A4<br>6H6<br>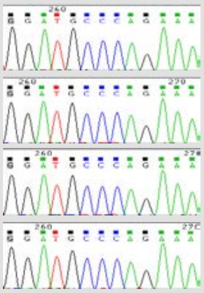

Supplementary Table 1 Analysis of putative off-target sites.

The sequence of the guide RNA targeting site TCGAGGGATGCCCAGAAACGGGG (PAM sequence in red) was used in a BLAST (Basic Local Alignment Search Tool) search for short sequences in the NCBI database against the human non-reduntant reference sequence. The BLAST search was performed to identify putative off target sites in other genes, which are summarized in the table (December 2016). The primer sequences are given for each PCR product generated at the different loci. PCR products were Sanger sequenced and no evidence for a putative unspecific editing at one of those loci was detected.

Supplementary Table 2 qPCR primer overview

Primer name	Sequence
HSPD1-F	GATGGAGAAGCTCTAAGTACACT
HSPD1-R	GCTGGTTCTTTCTATTGTCACCA
HPRT1-F	TGATAGATCCATTCCTATGACTGTAGA
HPRT1-R	AAGACATTCTTTCCAGTTAAAGTTGAG
GAPDH-F	CTGGGCTACACTGAGCACC
GAPDH-R	AAGTGGTCGTTGAGGGCAATG
MAP2_F	CGAAGCGCCAATGGATTCC
MAP2_R	TGAACTATCCTTGCAGACACCT
SYP-F	CCAATCAGATGTAGTCTGGTCAGT
SYP-R	AGGGGTGGAGACCTAGGGTA
DLG4-F	TCACAACCTCTTATTCCCAGCA
DLG4-R	CATGGCTGTGGGGTAGTAGTCG
NESTIN-F	CAGCGTTGGAACAGAGGTTGG
NESTIN-R	TGGCACAGGTGTCTCAAGGGTAG
SHANK1-F	CTCCCTGCGTTCCAAATCTA
SHANK1-R	GGCTGCTGCTCGTACTCC
SHANK2-F	CTTTGGATTCGTGCTTCGAG
SHANK2-R	CATCCACGGACTCCAGGTA
SHANK3-F	TTCCACGGACCAAGTCTGTA
SHANK3-R	GTCTTGCATCGAGGTGCTC
GRIA2_F	ACTGACACCCCACATCGAC
GRIA2_R	TCGAAAACTGGGAGCAGAAA
GRM1_F	ATGTCTCTGCAGTCCACACG
GRM1_R	CAGAATGGGCGATACAGAGG
GRIK2_F	TGGATATTCTCAAGGAACCACAC
GRIK2_R	TCACAGCAAATCTGAATGCAA
GABRB3_F	TGAGCTCCCGCAGTTCTC
GABRB3_R	CAGTGACAGTCGAGGATAGGC
TH-F	GCCCTACCAAGACCAGACGTA
TH-R	CGTGAGGCATAGCTCCTGA
